# A marine-derived fatty acid targets the cell membrane of Gram-positive bacteria

**DOI:** 10.1128/jb.00310-23

**Published:** 2023-10-31

**Authors:** Isha Upender, Olivia Yoshida, Anna Schrecengost, Hilary Ranson, Qihao Wu, David C. Rowley, Shreya Kishore, Claire Cywes, Eric L. Miller, Kristen E. Whalen

**Affiliations:** 1 Department of Biology, Haverford College, Haverford, Pennsylvania, USA; 2 Department of Biomedical and Pharmaceutical Sciences, University of Rhode Island, Kingston, Rhode Island, USA; 3 College of Pharmacy, University of Rhode Island, Kingston, Rhode Island, USA; Geisel School of Medicine at Dartmouth, Hanover, New Hampshire, USA

**Keywords:** Gram-positive bacteria, fatty acids, cell membranes, permeability

## Abstract

The rapid evolution of antibiotic resistance is shrinking our stockpile of commercially available antibiotics. Therefore, new antimicrobials with novel mechanisms of action (MoAs) are desperately needed. The free fatty acid, (*Z*)-13-methyltetra-4-decenoic acid ((*Z*)-4C-14:1), isolated from a marine sediment bacterium *Olleya marilimosa*, displays strong inhibition of Gram-positive pathogens with minimal cytotoxic effects to mammalian cells. Here, we applied a combination of experimental approaches to identify the mechanism by which (*Z*)-4C-14:1 kills the Gram-positive bacterium *Bacillus subtilis*. Using quick and cost-effective bacterial cytological profiling (BCP), we established the cytological signatures of 17 antibiotics representing 6 general classes of antibiotic MoAs. We used BCP to demonstrate that while (*Z*)-4C-14:1-treated *B. subtilis* cells display unique morphological features compared to other antibiotic classes, (*Z*)-4C-14:1 mode of action shares substantial overlap with the fast-acting antibiotic colistin specifically in Gram-positive cells and daptomycin, both of which target bacterial permeability by destroying the cell membrane and causing extensive cell surface alterations. To further determine if the cell membrane of *B. subtilis* was the target of (*Z*)-4C-14:1, we used cell membrane- and peptidoglycan-specific diagnostic stains to investigate bacterial permeability. Our results indicate that (*Z*)-4C-14:1 destabilizes the cell membrane by pore formation in Gram-positive bacteria and emphasize the importance of mining the marine environment for naturally occurring antibiotics.

**IMPORTANCE:**

With the lack of new antibiotics in the drug discovery pipeline, coupled with accelerated evolution of antibiotic resistance, new sources of antibiotics that target pathogens of clinical importance are paramount. Here, we use bacterial cytological profiling to identify the mechanism of action of the monounsaturated fatty acid (*Z*)-13-methyltetra-4-decenoic acid isolated from the marine bacterium *Olleya marilimosa* with antibacterial effects against Gram-positive bacteria. The fatty acid antibiotic was found to rapidly destabilize the cell membrane by pore formation and membrane aggregation in *Bacillus subtilis*, suggesting that this fatty acid may be a promising adjuvant used in combination to enhance antibiotic sensitivity.

## INTRODUCTION

The World Health Organization has estimated that 10 million people will perish annually by 2050 from drug-resistant infections impervious to many antibiotics (1), making the development of new treatments a priority. Bacterial resistance mechanisms alter antibiotic targets, reduce the intracellular antimicrobial concentration, and produce enzymes that deactivate the compound entirely, thereby limiting the lifespan of antibiotics (2). The rise of drug-resistant Gram-positive bacteria, specifically methicillin-resistant *Staphylococcus aureus*, poses severe human threats by evading many known classes of antibiotics including ß-lactams and antimicrobial peptides (3, 4). Compounding the threat of antibiotic resistance is the drought in the antibiotic discovery pipeline (5, 6) due to both the lack of biologically relevant chemical diversity in combination with synthetic approaches that have yet to supplant structural novelty of natural products (7). Therefore, searching for new antibacterial candidates with unique mechanisms of action (MoAs) is crucial to combat bacterial resistance mechanisms.

Recently, the discovery of new natural product-based antibiotics, teixobactin from soil (8) and darobactin from invertebrate symbionts (9), has reinvigorated antibiotic research in mining distinct environment niches for new chemical sources. In particular, the ocean serves as a diverse repository of untapped biological inventory whereby organisms have evolved the ability to synthesize a variety of potent chemical moieties resulting from competitive interactions between microbial life (10). Oceanic sediments represent a major global biome, harboring complex microbial communities likely structured by biologically active natural products as revealed by environmental metabolomic profiling (11). Even within an apparent homogeneous environment of sediment exists complex chemical landscapes associated with marine microbial communities, and paired metagenomic sequencing efforts hint at the vast, yet undescribed, natural product diversity awaiting discovery (12).

Here, we describe the isolation, synthesis, and characterization of (*Z*)-13-methyltetra-4-decenoic acid ((*Z*)-4C-14:1), a *cis*-monounsaturated novel free fatty acid (FFA) derived from the marine, Gram-negative bacterium *Olleya marilimosa* (A414) isolated from a marine sediment. Culture-based assays demonstrated that (*Z*)-4C-14:1 is a rapid-acting compound most effective against Gram-positive bacteria *Bacillus subtilis*, *S. aureus*, and *Enterococcus faecalis*. Moreover, significant cytotoxicity was observed in human liver (HepG2) cells only at concentrations above 800 µM. To elucidate the MoA of (*Z*)-4C-14:1, we employed fluorescence-based microscopy to discriminate between different MoAs of known antibiotics and our marine compound by analyzing the cell morphological profiles upon antibiotic treatment using bacterial cytological profiling (BCP) (13). BCP has proven effective in MoA studies of antibiotics (14), complex mixtures (15), and synergistically acting natural products (16). We show that (*Z*)-4C-14:1 has a unique cytological profile that clusters with those antibiotics targeting cell permeability, with specific overlap with antibiotics that inhibit cell membrane functioning. Diagnostic fluorescence staining of *B. subtilis* cells exposed to (*Z*)-4C-14:1 confirmed the cell membrane to be significantly compromised while the peptidoglycan layer was still contiguous, suggesting that (*Z*)-4C-14:1 may have detergent-like capabilities. Together, these findings support further development of (*Z*)-4C-14:1 as a promising candidate to combat Gram-positive bacterial infections when used in combination with other classes of antibiotics to enhance susceptibility.

## RESULTS

### Identification and activity testing of (*Z*)-4C-14:1

Using the bacterial susceptibility assay, which identifies isolates capable of reducing an antibiotic’s minimum inhibitory concentration (MIC) greater than or equal to fourfold, extract from the isolate A414 was found to potentiate the activity of erythromycin when tested against *Escherichia coli* multidrug resistant (MDR) strain MG1655 ΔBC/pXYM (17), overexpressing the efflux transporter MexXY-OprM, in the *p*-iodonitrotetrazolium chloride (INT) assay as described in Whalen et al. (18). Strain A414 was subsequently identified as *O. marilimosa* based on 16S rRNA gene sequencing (99.64% identity *O. marilimosa* strain Y23, NCBI accession number MN746117.1). A total of 30 L of A414 bacterial culture was grown yielding 4.5 g of crude organic extract, which was subjected to bioassay-guided fractionation through three rounds of chemical fractionation [i.e., silica column, SPE/ENVI-18 Sep-Pak, reverse-phase high-performance liquid chromatography (HPLC)] as described in Fig. S1, with each semi-purified fraction being tested for activity in the bacterial susceptibility assay until a single pure compound was identified. The resulting active compound was isolated as a clear oil. The HRESIMS analysis established the molecular formula as C_15_H_28_O_2_ (*m/z* 239.2011, [M–H]^−^ calcd. 239.2017). The ^13^C nuclear magnetic resonance (NMR) spectrum, combined with the HSQC experiment (Fig. S2) and the degree of unsaturation of 2, determined by the molecular formula, indicated that the compound is a fatty acid with one double bond. The ^1^H–^1^H COSY correlations of H_2_-2 (*δ*_H_ 2.22)/H_2_-3 (*δ*_H_ 2.22)/H-4 (*δ*_H_ 5.32)/H-5 (*δ*_H_ 5.35)/H_2_-6 (*δ*_H_ 2.00), along with the key HMBC correlations from H_2_-3 to C-1 (*δ*_C_ 173.9) and from H-4 to C-2 (*δ*_C_ 33.8), allowed for the location of the double bond at C-4/C-5 (Fig. S2). The double bond geometry at C-4/C-5 was assigned as *Z* by observing that the pattern of the olefinic proton signals in the ^1^H NMR spectrum of the isolate was similar to that of methyl oleate (*Z*) rather than methyl elaidate (*E*) (19). Furthermore, the methyl signals at *δ*_H_ 0.84 (6H, d, *J* = 6.5 Hz) as well as the molecular formula suggested the compound to be (*Z*)-4C-14:1. Only 8.9 mg of this compound was isolated from the initial 4.5 g of crude extract; therefore, (*Z*)-4C-14:1 was synthesized (Fig. S3) yielding 544 mg of a pure compound and was determined to be identical to the naturally occurring compound by NMR spectroscopy (Fig. S4 and S5).

**Fig 1:**
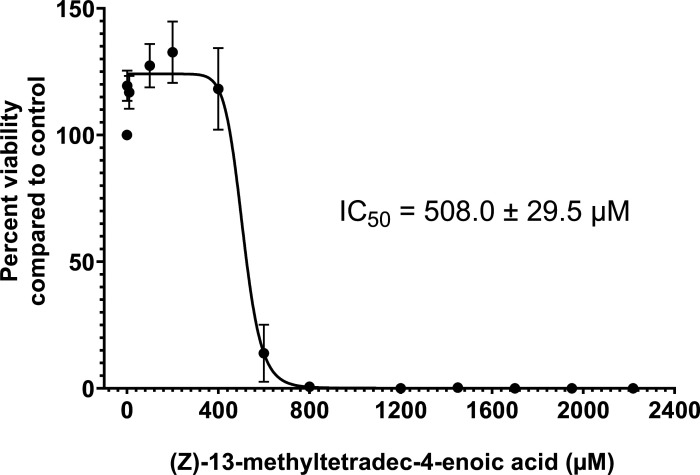
Evaluation of (*Z*)-4C-14:1 cytotoxicity in the HepG2 cell line. HepG2 cells were treated with (*Z*)-4C-14:1 for 48 h, and the relative cytotoxicity was determined by luminescence using the CellTiter-Glo 2.0 (ATP) assay. Percent viability was calculated as a percentage of control cells (0.9% DMSO only). The mean ± SEM of five replicates are shown.

Bacterial susceptibility screening with the isolated natural compound indicated that the MICs against *E. coli* MDR strain MG1655 ΔBC/pXYM overexpressing the efflux transporter MexXY-OprM (17) and the methicillin-sensitive clinical isolate *S. aureus* (DMS 1104) were 75 and 30 µg/mL, respectively. Due to the limited amount of isolated natural compound, all subsequent experimentation was performed with the synthetic version of (*Z*)-4C-14:1. Next, we examined the biological activity of (*Z*)-4C-14:1 against a panel of different pathogens. (*Z*)-4C-14:1 demonstrated activity exclusively against Gram-positive bacteria (Table 1). Additionally, (*Z*)-4C-14:1 was evaluated for its cytotoxicity against human liver HepG2 cells using a luminescence-based cell viability assay that detects the presence of ATP in viable cell populations. (*Z*)-4C-14:1 only exhibited substantial cytotoxic effects at concentrations above 800-µM (192.3 µg/mL) final concentration. The average of five independent assays was used to calculate an IC_50_ value of 508.0 ± 29.5 µM (Fig. 1).

**TABLE 1:** MIC of (*Z*)-4C-14:1 compared to tetracycline against select pathogens in CAMHB^*a*^

Strain	MIC of synthetic (*Z*)-4C-14:1	MIC of tetracycline
*B. subtilis* ATCC 23857	32 µg/mL	2 µg/mL
*E. faecalis* ATCC 29212	28 µg/mL	16 µg/mL
*E. coli* ATCC 25922	>128 µg/mL	0.5 µg/mL
*P. aeruginosa* ATCC 27853	>128 µg/mL	8 µg/mL
*S. aureus* ATCC 29213	28 µg/mL	0.125 µg/mL
*S. pneumoniae* ATCC 49619	>128 µg/mL	0.06 µg/mL

^*a*^ MIC determinations were performed in triplicate for each pathogen.

### Evaluation of (*Z*)-4C-14:1 by BCP

**Fig 2:**
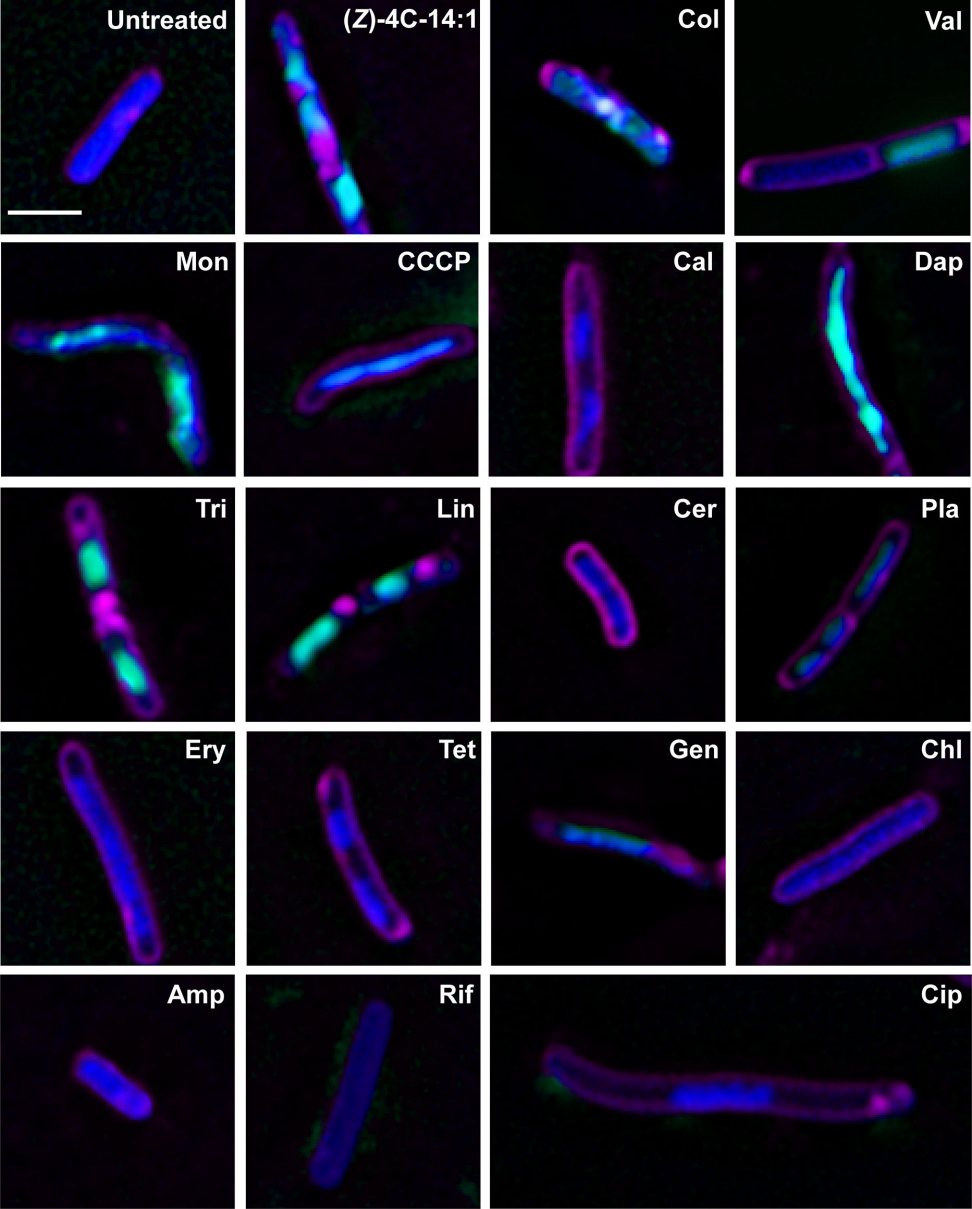
Composite image of bacterial cell morphology with antibiotics and (*Z*)-4C-14:1. *B. subtilis* cytological profiling of untreated, (*Z*)-4C-14:1 (2 h), and known antibiotics that affect the membrane: colistin (Col, 0.5 h), valinomycin (Val, 2 h), monactin (Mon, 3 h), CCCP (4 h), calcimycin (Cal, 2 h), daptomycin (Dap, 2 h), triclosan (Tri, 2 h), and linoleic acid (Lin, 2 h); fatty acid synthesis: cerulenin (Cer, 4 h) and platensimycin (Pla, 6 h); protein synthesis: erythromycin (Ery, 6 h), tetracycline (Tet, 5.5 h), gentamicin (Gen, 5.5 h), and chloramphenicol (Chl, 6 h); cell wall synthesis: ampicillin (Amp, 5.5 h); transcription: rifampicin (Rif, 5.5 h); and DNA replication: ciprofloxacin (Cip, 6 h). Bacterial cells were treated with each antibiotic at 5× MIC, with the exception of valinomycin (2× MIC), and then stained with FM4-64 (magenta), DAPI (blue), and SYTOX Green (green). Scale bar represents 2 µm.

**Fig 3:**
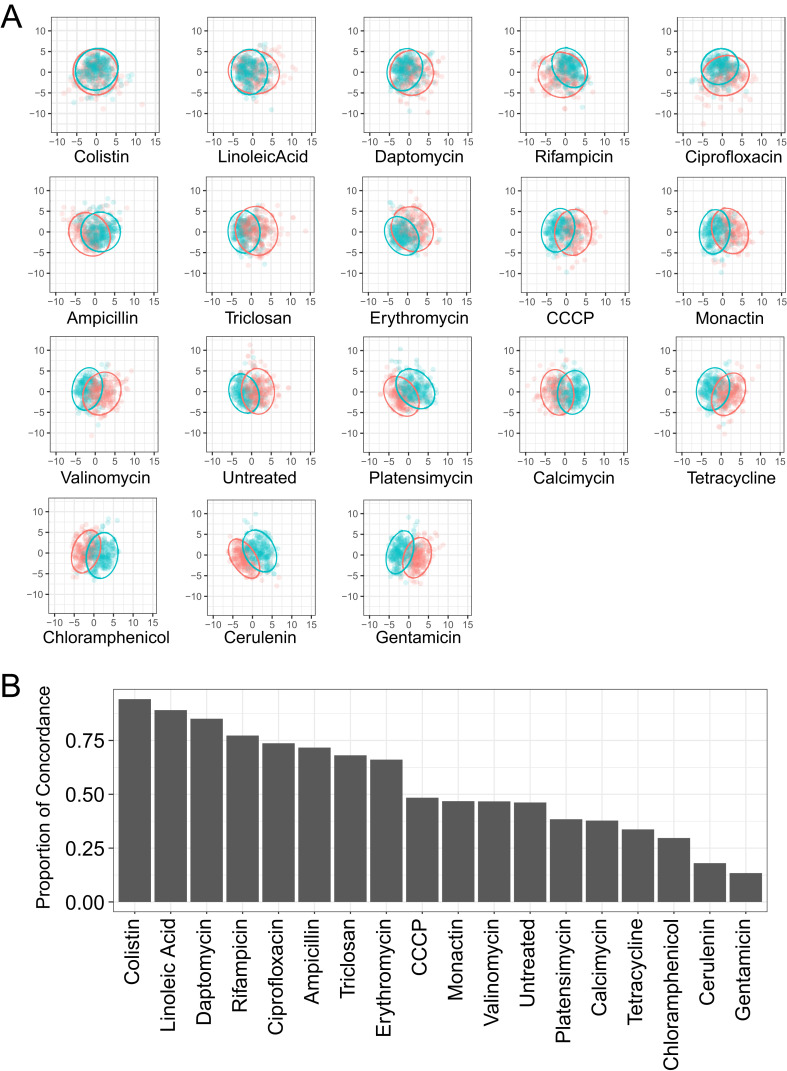
Pairwise PCA of *B. subtilis* cytological profiles. (**A**) Pairwise PCA showing the first two principal components of the cytological profiles for all cells from (*Z*)-4C-14:1-treated *B. subtilis* to antibiotic-treated *B. subtilis*. 95% confidence ellipses for (*Z*)-4C-14:1-treated *B. subtilis* are in blue and for the other treatment in orange. (**B**) Proportion of concordance for (*Z*)-4C-14:1-treated *B. subtilis* with antibiotic-treated *B. subtilis*. Using the pairwise PCAs, we calculated concordance as the proportion of cells within either 95% confidence ellipse that were within both individual 95% confidence ellipses for the two treatments.

*B. subtilis* cells were used in BCP experiments due to the dynamic change in cell morphology upon exposures to various classes of antibiotics (20). *B. subtilis* was treated with (*Z*)-4C-14:1 and antibiotics across six diverse inhibition categories (Fig. 2): cell wall inhibition (ampicillin and linoleic acid), ionophores [calcimycin, carbonyl cyanide 3-chlorophenylhydrazone (CCCP), monactin, and valinomycin], protein synthesis inhibition (chloramphenicol, erythromycin, gentamicin, and tetracycline), DNA/RNA inhibition (ciprofloxacin and rifampicin), fatty acid synthesis inhibition (cerulenin, platensimycin, and triclosan), and disruption of cytoplasmic membrane (daptomycin and colistin in Gram-positive cells specifically). A total of 39 cytological parameters were measured for each cell within each antibiotic treatment from four independent experiments. Variable reduction was performed using a principal component analysis (PCA) on a per-cell basis from (*Z*)-4C-14:1 treatments and from each other antibiotic with known MoAs in pairwise comparisons (Fig. 3A). Concordance of 95% confidence ellipses indicated that (*Z*)-4C-14:1-treated *B. subtilis* was morphologically most similar to antibiotics colistin, linoleic acid, and daptomycin, all of which target bacterial permeability, albeit through different mechanisms (Fig. 3B).

### Loss of membrane integrity

**Fig 4:**
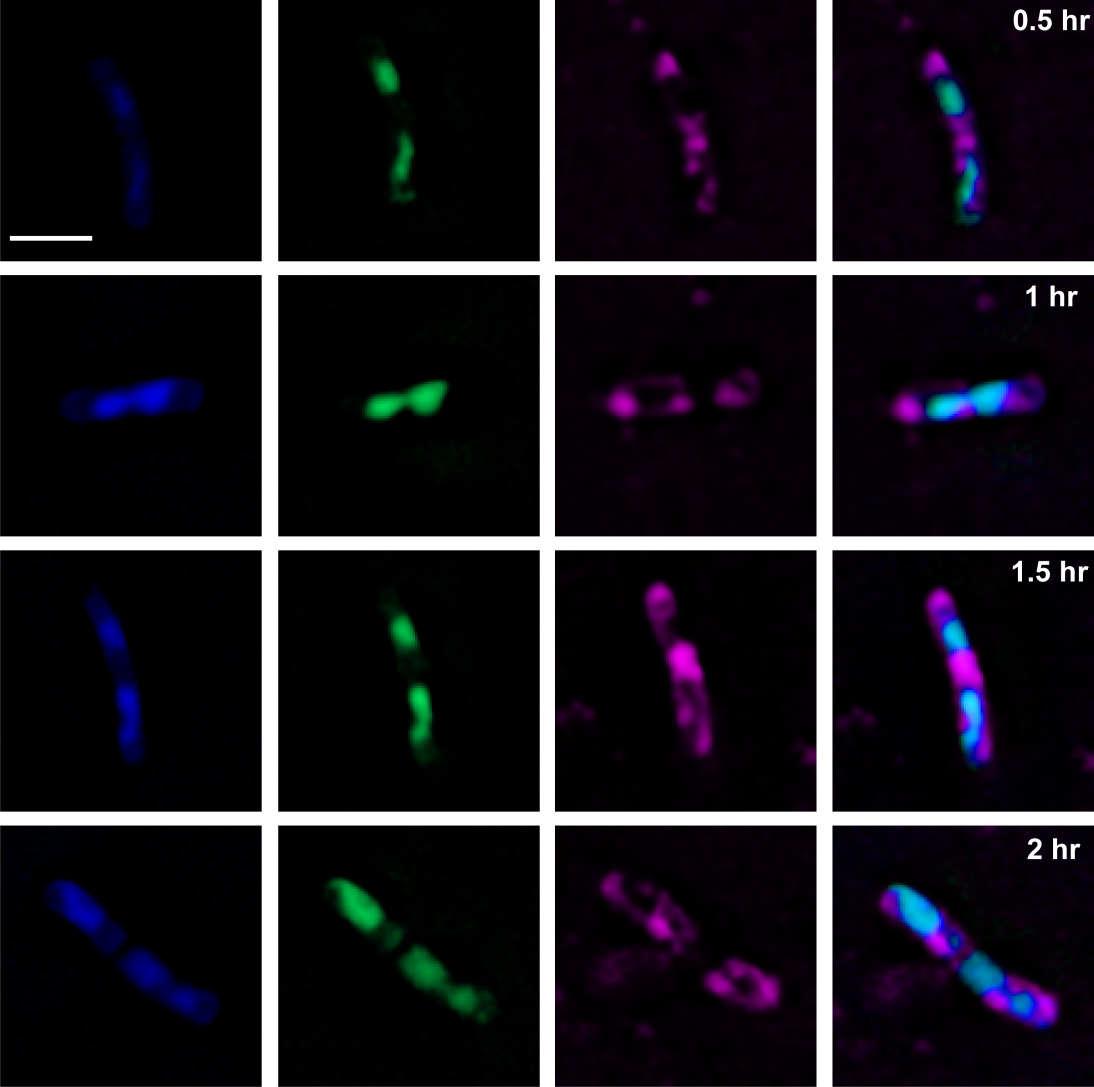
Composite image of the (*Z*)-4C-14:1 compound over time. *B. subtilis* cells treated with (*Z*)-4C-14:1 at 5× MIC every 30 min. Bacterial cells were then stained with FM4-64 (magenta), DAPI (blue), and SYTOX Green (green). Scale bar represents 2 µm.

**Fig 5:**
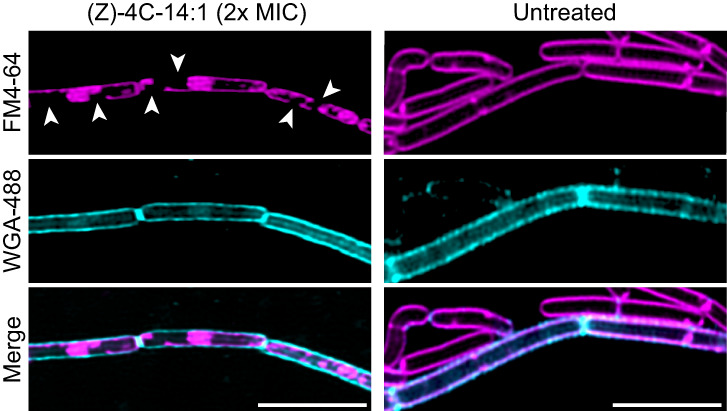
Specific labeling of the cell membrane and peptidoglycan layer of untreated and (*Z*)-4C-14:1-treated *B. subtilis. B. subtilis* cells were exposed to (*Z*)-4C-14:1 at 2× MIC or a solvent control for 2 h and then visualized live. Cell membranes were stained with 6 µg/mL of FM4-64 (magenta), and the peptidoglycan layer was stained with 100 µg/mL of *N*-acetylglucosamine (GlcNAc)-specific WGA lectin conjugated to Alexa Fluor 488 (cerulean). White arrow heads indicate loss of cell membrane integrity without a corresponding loss of peptidoglycan layer integrity in cells treated with (*Z*)-4C-14:1. Scale bar represents 5 µm.

To examine the rapidity of morphological changes in cells exposed to (*Z*)-4C-14:1, *B. subtilis* cells were treated with (*Z*)-4C-14:1 at 5× MIC and visualized in 30-min intervals for 2 h using diagnostic fluorescence staining. Membrane disruption/aggregation and SYTOX staining were detected in (*Z*)-4C-14:1-treated cells as early as 30 min (Fig. 4), which mirrors the onset of morphological changes seen with colistin at 30-min post-exposure and linoleic acid at 2-h post-exposure, respectively. Reports indicate that polymyxins, like colistin, can get through the peptidoglycan layer of Gram-positive cells and destroy the integrity of the cytoplasmic membrane (21), resulting in leakage of cellular contents (22). To confirm if (*Z*)-4C-14:1 also targets the cell membrane, we performed diagnostic fluorescence microscopy to assess the structural integrity of both the peptidoglycan layer stained with wheat germ agglutinin (WGA)-Alexa Fluor 488 and the cell membrane stained with FM4-64 in untreated and (*Z*)-4C-14:1-treated *B. subtilis*. Cells exposed to (*Z*)-4C-14:1 at 2× MIC for 2 h were observed to show membrane collapse via the formation of membrane pores and membrane aggregates, which were absent in untreated cells (Fig. 5). Additionally, even though the loss of membrane integrity was observed in (*Z*)-4C-14:1-treated cells, the peptidoglycan layer remained intact, indicating that (*Z*)-4C-14:1 initially targets the cell membrane of Gram-positive cells.

## DISCUSSION

In particular, FFAs offer considerable antibiotic potential due to their broad activity spectrums effective against various Gram-positive and Gram-negative pathogens (23, 24) and difficulties in bacterial resistance development (25). Due to their amphipathic properties, antibacterial FFAs are capable of destabilizing cell membranes, disrupting both the electron transport chain, and uncoupling oxidative phosphorylation (23, 25). Here, we describe the identification of (*Z*)-4C-14:1 from a marine bacterium with activity against select Gram-positive pathogens. The MIC of (*Z*)-4C-14:1 against a methicillin-sensitive clinical isolate of *S. aureus* was determined to be 30 µg/mL, which is well below the MICs for comparable fatty acids including lauric acid (C12:0) at 400 µg/mL, myristic acid (C14:0) at 1,600 µg/mL, and palmitic acid (C16:0) at >1,600 µg/mL (26). Through the use of BCP in combination with diagnostic fluorescence microscopy, we identified (*Z*)-4C-14:1 as a rapid-acting antibiotic that specifically induces pore formation in bacterial cell membranes, resulting in destabilization.

(*Z*)-4C-14:1 is a *cis*-monounsaturated long-chain fatty acid compound isolated from marine *O. marilimosa* within the family Flavobacteriaceae. The first report of this branched-chain monoenoic fatty acid 13-methyl-4-14:1 was described from a Black Sea sponge, *Dysidea fragilis* (27), with a subsequent description of its isolation from an additional sponge (28); however, the biological activity of the compound was never explored. Marine sponges contain diverse microbial communities that can account for up to 40% of the host volume (29), and it is now recognized that associated bacteria are typically the source of these C_15_-C_19_ fatty acids within sponge tissues (30). Consistent with a bacterial origin of sponge-associated, unsaturated fatty acids was the isolation of additional monounsaturated *iso*-branched fatty acids identified from members of the Cytophaga-Flavobacterium-Bacteroides group of bacteria (31) and the report of (*Z*)-4C-14:1 isolation from *Flavobacterium* within the family Flavobacteriaceae (32).

Concordance comparison of PCA results allowed us to narrow down the possible MoA of (*Z*)-4C-14:1 to bacterial permeability, with the highest similarities to antibiotics colistin (polymyxin E), linoleic acid, and daptomycin, albeit affecting bacterial permeability in different ways. Colistin and (*Z*)-4C-14:1 are both fast-acting antibiotics that showed similar rapid membrane aggregation/blebbing morphology changes on *B. subtilis*-treated cells within 30 min of application. Colistin is most notable for its ability to target the lipopolysaccharide component of the outer membrane of Gram-negative bacteria by competing with divalent cations to destabilize the outer membrane, resulting in permeability (21). In Gram-positive bacteria that lack an outer membrane, once polymyxins are able to penetrate through the peptidoglycan layer, these compounds destroy the integrity of the membrane resulting in cell leakage (21, 22). Initial investigations into linoleic acid’s MoA describe the compound’s action as a surfactant-like increase in membrane permeability (33); however, additional work in *S. aureus* suggests that linoleic acid can alter the expression of genes for peptidoglycan synthesis (34) and inhibit fatty acid synthesis (35), both of which can impact bacterial permeability. For daptomycin, there are two general hypotheses as to this antibiotic’s MoA. The first suggests that aggregates of daptomycin form oligomeric pore structures in the membrane, resulting in ion leakage and the loss of membrane potential (36). A second hypothesis suggests that daptomycin asserts a lipid-extracting effect whereby compound insertion into the membrane causes rapid aggregation of the lipid on the membrane surface, the eventual release of lipid clusters from the membrane, and the formation of transient water pores that could explain the ion leakage observation seen previously (36). Because all three antibiotics with high concordance to (*Z*)-4C-14:1 have been shown to permeabilize bacterial cells, we specifically interrogated the structural integrity of both the peptidoglycan layer and membrane by fluorescence microscopy of *B. subtilis* cells exposed to (*Z*)-4C-14:1. We observed significant pore formation after 2-h exposure at 2× MIC, accompanied with membrane aggregation and no apparent loss of peptidoglycan integrity, indicating that (*Z*)-4C-14:1 initially targets phospholipid membrane stabilization. Our result is consistent with recent findings investigating various natural long-chain unsaturated fatty acids found to induce concentration-dependent membrane remodeling behavior *in vitro* (37). Finally, we did not see appreciable antibiotic activity of (*Z*)-4C-14:1 against the Gram-positive *Streptococcus pneumoniae*, which we postulate is likely as a result of the physical thickness of the polysaccharide capsule that may prevent physical contact of (*Z*)-4C-14:1 with the membrane.

*Cis*-arrangement (38, 39), iso-branching (40), and long-chained unsaturated fatty acids (41) have a long history of antimicrobial activity against Gram-positive bacteria (23). Specifically, unsaturated fatty acids with long chains consisting of 12 or more carbons demonstrate exceptional activity against Gram-positive bacteria (42, 43). Moreover, unsaturated fatty acids have been shown to inhibit the growth of both Gram-negative and Gram-positive bacteria (reviewed in (24)). Interestingly, (*Z*)-4C-14:1 was originally isolated in a screen for antibiotic adjuvants targeting Gram-negative bacteria overexpressing resistance nodulation cell division (RND) transporters. This assay functions by screening complex chemical mixtures of bacterial exudates in *E. coli* overexpressing RND pumps grown in a background of antibiotic at one-fourth of their MIC concentration. This finding suggests that (*Z*)-4C-14:1 could exhibit synergistic effects with other antibiotics by permeabilizing the outer membrane of Gram-negative bacteria. This strategy of combining fatty acid adjuvants with antibiotics has been shown to be effective in overcoming bacterial drug resistance and inhibiting biofilm formation (9, 26, 44 – 51). For example, when the synergistic activities of 30 FFAs and 11 antibiotics were investigated against MRSA, the monounsaturated myristoleic acid (C14:1) most enhanced the bactericidal activities of four aminoglycoside antibiotics synergistically, including significantly decreasing biofilm formation by *S. aureus* (46).

In summary, the broad spectrum of activity of unsaturated FFAs, their low eukaryotic cytotoxicity, and lack of FFA resistance mutants make them attractive agents for further exploration for treating drug-resistant pathogens. Marine organisms are a rich source of novel FFAs with proven biomedical potential (52). Furthermore, the essential function of the cell membrane makes it a robust target for developing new antibacterial therapies. The use of membrane-disrupting unsaturated FFAs in synergy with approved therapeutics is a promising strategy to combat bacterial infections.

## MATERIALS AND METHODS

### General experimental procedures

NMR spectroscopies including 1D (^1^H and ^13^C) and 2D (COSY, HSQC, and HMBC) were performed on a 500-MHz Varian Inova NMR spectrometer, and the chemical shifts were recorded as *d* values (ppm) referenced to solvent residual signals [DMSO-*d*_6_ (*δ*_H_ 2.50 ppm, *δ*_C_ 39.520 ppm)]. Active HPLC fractions underwent untargeted chemical profiling on an Agilent G6125BW liquid chromatography-mass spectrometer (LC-MS) equipped with an Agilent 1260 Infinity II series HPLC with a Phenomenex Kinetex 2.6 µm, C18, 100 Å, LC column (150 × 2.1 mM) as the stationary phase. Spectra were collected in both positive ((+)-HRESI) and negative ((−)-HRESI) ionization modes. All HPLC-MS experiments used a flow rate of 0.2 mL/min. The instrument was equipped with OpenLAB CDS ChemStation Edition (Rev. C.01.08) software. Antibiotic isolation was accomplished using vacuum liquid chromatography with silica gel, pore size 60 Å, particle size 40–60 µm (Sigma-Aldrich). All solvents used throughout the project were OPTIMA grade (Fisher Scientific). Semipreparative HPLC was carried out on an Agilent 1260 Infinity II series equipped with an autosampler, a diode array detector, a quaternary pump, and a 96-well plate fraction collector with a Phenomenex Kinetex 5 µm C18, 100 Å column (150 × 10 mM) as the stationary phase. All semipreparative HPLC experiments used a flow rate of 4 mL/min.

### Microbial isolation and identification

Marine bacterial isolate A414 was part of the Mincer Culture Collection housed at the Woods Hole Oceanographic Institution and originally isolated for a sediment sample from Clayoquot Sound, British Columbia, Canada onto a tryptone seawater (TSW) agar (TSW: 1-g/L tryptone; 1 L of 75:25 natural seawater: Milli-Q water; 15-g agar) plate and cultivated at 23℃ for 3 days. A single yellow colony was transferred to a fresh TSW plate to obtain a pure isolate of strain A414.

Genomic DNA was isolated from a bacterial pellet of A414 with the Qiagen DNeasy Blood and Tissue Kit (Qiagen, Valencia, CA, USA) following the manufacturer protocol with an enzymatic lysis buffer containing 50-mM Tris-Cl, 10-mM sodium-EDTA, 1.2% Triton X-100, and lysozyme at a final concentration of 20 mg/mL. The bacterial small subunit rRNA gene was amplified using PCR with oligonucleotide primers 27F (5′- AGAGTTTGATCMTGGCTCAG-3′) and 1492R (5′-TACGGYTACCTTGTTACGACTT-3′). A 50-µL PCR reaction contained 1.25 U of GoTaq(R) G2 Flexi DNA polymerase (Promega, Fitchburg, WI, USA), 1× GoTaq Flexi Buffer, 1.25-mM MgCl_2_, 200 µM of each dNTP, 200 nM of each primer, and 250 ng of the genomic template and was performed in a MyCycler thermal cycler (Bio-Rad Laboratories, Hercules, CA, USA). Amplification of PCR products was carried out according to the GoTaq(R) Flexi Kit, and cycling parameters were as follows: 95℃ for 2 min; 40 cycles of 95℃ for 20 s, 54℃ for 30 s, and 72℃ for 1.5 min; and 1 cycle of 72℃ for 5 min. The amplification product was subjected to gel electrophoresis in 1% agarose gels and purified using the QIAquick PCR Purification Kit (Promega, Madison, WI, USA). The product was sequenced in a single direction using the Sanger method by Eurofin MWG Operon Biotech. Sequences were manually inspected using Sequencher 4.8 (Gene Codes, Ann Arbor, MI, USA), and ambiguous nucleotides on the ends of the sequence were excluded from further analysis. Phylogenetic analysis indicated that the isolated strain A414 was *O. marilimosa* on the basis of 99.85% identity in the 16S rRNA gene sequence (GenBank accession number OP950677). The pure culture was cryopreserved in 10% sterile DMSO and stored at −80℃ until use.

### Bacterial culture and extract production

A starter culture of A414 was prepared by inoculating 10 mL of TSW media with 100 µL of cryopreserved stock and grown for 3 days at 23℃ at 100 rpm. To scale up bacterial growth conditions, large batch cultures consisted of inoculating a 1.5-L TSY (1-g/L tryptone, 1-g/L yeast extract, and 1.5-L of 75:25 natural seawater: Milli-Q water) culture in a 2-L Fernbach flask with 2 mL of starter culture. This large batch culture was grown at 18℃ at 100 rpm for 8 days. Twenty-four hours prior to culture filtration (Day 7), 20 mL of a 1:1 mixture of sterile, washed Amberlite XAD-7 and XAD-16 resin was added to the cultures. On Day 8, the resin was filtered under vacuum, desalted by rinsing in 300-mL Milli-Q water, and dried overnight at room temperature before storing at −20℃ until use. The secreted metabolites were eluted from the resin first in 100 mL of (1:1) methanol/dichloromethane, followed by 100 mL of methanol/20 mL of XAD resin. Both organic fractions were combined and dried under vacuum centrifugation (Thermo Savant) and stored at −20℃ until bacterial susceptibility assays.

### Isolation of (*Z*)-4C-14:1

In sum, 30 L of A414 culture was processed as described above and it yielded 4.5 g of crude extract (Fig. S1). The crude extract was subjected to silica gel column chromatography with a step gradient of 100% isooctane, 4:1 isooctane/ethyl acetate, 3:2 isooctane/ethyl acetate, 2:3 isooctane/ethyl acetate, 1:4 isooctane/ethyl acetate, 100% ethyl acetate, 1:1 ethyl acetate/methanol, and 100% methanol, yielding eight fractions. Active constituents, determined via the INT assay, eluted between 4:1 and 2:3 isooctane/ethyl acetate (fractions 2–4) were combined totaling 44.7 mg and further fractionated by SPE/ENVI-18 Sep-Pak (Supelco, Cat No. 57138) with a step gradient of 95:5 water/acetonitrile, 2:3 water acetonitrile, 5:95 water acetonitrile, and 100% acetone. All solvents with the exception of acetone were acidified with 0.1% formic acid. Active constituents eluted in 2:3 water/acetonitrile and 5:95 water/acetonitrile (fractions 3 and 4). Active fractions were combined and totaled 33.2 mg and were further separated using a gradient of acidified (0.1% formic acid) methanol and water by semipreparative HPLC using an Agilent 1260 Infinity II series equipped with a Phenomenex Kinetex C18 (150 × 10 mM) LC column as the stationary phase with a flow rate of 4 mL/min. Chromatography methods were as follows: hold for 4 min at 4:1 water/methanol, ramp up to 95% water/methanol over 1 min, hold at 95% methanol for 7 min, then immediately revert to starting conditions, and hold for 8 min. The active compound, totaling 8.9 mg, eluted in HPLC fraction 3 at 95% methanol between 9 and 9.3 min.

The active HPLC fraction was resuspended at 2 mg/mL in 4:1 water/methanol and profiled on an Agilent G6125BW LC-MS equipped with an Agilent 1260 Infinity II series HPLC with a Phenomenex Kinetex 2.6 µm, C18, 100 Å, (150 × 2.1 mM) LC column at a flow rate of 0.2 mL/min using the following solvent scheme: hold for 4 min at 5% methanol, then ramp to 95% over 1 min, hold at 95% methanol for 7 min, and immediately revert to starting conditions of 5% methanol for 8 min. Targeted MS/MS analysis was performed on the active A414 HPLC sample with the same solvent scheme used for LC-MS described above. LC-MS and MS/MS data were analyzed using MassHunter B.07.00. Compounds were identified after blank subtraction using the Molecular Feature Extractor tool and then filtering by peak area with absolute area ≥ 10,000 counts. Potential molecular formulas were determined using the generate formulas from compounds tool. For targeted MS/MS analysis, compounds were found by the targeted MS/MS feature with the Agile 2 algorithm as the integrator selection, with the parameter max peak width = 0.25 min and filtered by absolute peak area ≥ 10,000 counts. ^1^H NMR (500 MHz, DMSO-*d*_6_): *δ* 12.0 (brs, 1H), 5.32–5.35 (m, 2H), 2.22 (m, 4H), 2.00 (m, 2H), 1.50 (m, 1H), 1.25 (m, 10H), 1.14 (m, 2H), and 0.84 (d, *J* = 6.5 Hz, 6H). ^13^C NMR (125 MHz, DMSO-*d*_6_): *δ* 173.9, 130.5, 128.1, 38.4, 33.8, 29.6, 29.0, 28.9, 28.6, 27.4, 26.8, 26.5, 22.5, 22.4 (Fig. S2).

### Bacterial susceptibility determinations with isolated (*Z*)-4C-14:1

Initially, the crude extract from A414 was screened in a whole-cell assay described in Whalen et al. (18) against *E. coli* strains engineered to overexpress RND transporters with the aim of identifying either an efflux pump inhibitor or antimicrobial compound(s). This bacterial susceptibility assay was used to identify marine microbial isolates capable of reducing antibiotic MICs greater than or equal to fourfold in three strains overexpressing three archetype RND transporters (AcrAB-TolC, MexAB-OprM, and MexXY-OprM) common in Gram-negative pathogens. The crude extract from A414 was tested in duplicate at 0.25 mg/mL and visualized by rapid *p*-iodonitrotetrazolium (INT) chloride colorimetric assay in 96-well microtiter plates in a final volume of 200 µL. Test *E. coli* strains were grown in the presence of the extract and either chloramphenicol or erythromycin at one-fourth of their MICs. An extract was considered active if it was able to reduce the MIC of the co-administered antibiotic at least fourfold. The extract from A414 was chemically fractionated as described above, and at each step, the semi-purified fractions and final pure compound were screened in the whole-cell assay against *E. coli* for activity.

Initially, the MIC of the isolated (*Z*)-4C-14:1 was determined for *S. aureus* (DSM 1104), a methicillin-sensitive clinical isolate. The MIC was determined by broth microdilution in Luria Broth (LB) medium as previously described (53). *S. aureus* (DSM 1104) was obtained from the American Type Culture Collection (ATCC 25923).

### Synthesis of (*Z*)-4C-14:1

Synthesis of (*Z*)-4C-14:1 (Fig. S3) was prepared from a solution of 5-bromopentanol in anhydrous THF-added dropwise to a dry-ice/acetone bath-cooled solution of isopentylmagnesium bromide. A solution of Li_2_Cu_2_Cl_4_ (0.1 M in THF) was added following the complete addition of the alcohol. The resulting brown suspension was cooled once again in a dry-ice/acetone bath, and the reaction was slowly quenched by the addition of saturated aqueous NH_4_Cl. The mixture was concentrated to remove the THF and then extracted with EtOAc. The combined extracts were dried (MgSO_4_), and the drying agent was removed by filtration. Silica gel was added to the filtrate, and the mixture was concentrated to dryness under reduced pressure. Flash column chromatography (RediSepR_f_, SiO_2_, 100% hexane → 30% EtOAc in hexane, visualizing fractions with KMnO_4_ stain) gave the product as a pale clear liquid (11 g, 50%). ^1^H NMR (400 MHz, CDCl_3_): *δ* 3.62 (t, *J* = 7.2 Hz, 2H), 1.54 (m, *J* = 6.8 Hz, 2H), 1.51 (m, *J* = 6.8 Hz, 2H), 1.30–1.25 (m, 8H), 1.14 (m, 2H), and 0.84 (d, *J* = 6.8 Hz, 6H). ^13^C NMR (125 MHz, CDCl_3_): *δ* 63.1, 39.0, 32.8, 29.8, 29.4, 27.9, 27.3, 25.7, and 22.6. GCMS: *m/z* = 158.3.

In step 2, a solution of triphenylphosphine in anhydrous CH_2_Cl_2_ was added dropwise over 30 min to an ice-H_2_O bath-cooled solution of the alcohol and CBr_4_ in anhydrous CH_2_Cl_2_. The reaction was allowed to warm to room temperature as the cooling bath melted. Stirring was continued for 3 h, and the reaction was analyzed by TLC (10% EtOAc in hexane, KMnO_4_ stain) indicating complete consumption of the starting material. The clear, pale solution was concentrated to dryness under reduced pressure. The residue was triturated with hexane, then supernatants were combined with silica gel, and the mixture was concentrated to dryness under reduced pressure. Flash column chromatography (RediSepR_f_, SiO_2_, 100% hexane, fractions visualized with PMA stain) gave the product as a clear, colorless oil. ^1^H NMR (400 MHz, CDCl_3_): *δ* 3.93 (t, *J* = 7.6 Hz, 2H), 1.84 (m, *J* = 7.2 Hz, 2H), 1.48 (m, *J* = 7.2 Hz, 1H), 1.38 (m, 2H), 1.26 (m, 6H), and 1.14 (m, 2H). This material was used in the next step with no further characterization.

In step 3, a solution of n-BuLi was added dropwise over 10 min to a dry-ice/acetone bath-cooled solution of 4-pentynol and HMPA in anhydrous THF. The resulting mixture was allowed to stir at that temperature for 30 min. After that period of time, a solution of the bromide in THF was added dropwise over 5 min. The reaction was allowed to warm to room temperature as the cooling bath melted. Stirring was continued overnight. The resulting orange solution was quenched by the addition of saturated aqueous NH_4_Cl. The mixture was extracted with EtOAc. Silica gel was to the combined extracts, and the mixture was concentrated to dryness under reduced pressure. Flash column chromatography (RediSepR_f_, SiO_2_, 100% hexane → 20% EtOAc in hexane, visualizing fractions with KMnO_4_) gave the product as a pale transparent oil (2.1 g, 36%). ^1^H NMR (400 MHz, CDCl_3_): *δ* 3.71 (t, *J* = 6.0 Hz, 2H), 2.25 (m, 2H), 2.11 (m, 2H), 1.71 (m, *J* = 6.0 Hz, 2H), 1.62 (bs, 1H), 1.45 (m, *J* = 6.8 Hz, 1H), 1.41 (m, 2H), 1.30 (m, 2H), 1.28 (m, 8H), 1.12 (m, 2H), 1.12 (m, 2H), and 0.84 (d, *J* = 6.0 Hz, 6H). ^13^C NMR (100 MHz, CDCl_3_): *δ* 81.1, 79.2, 62.0, 39.0, 31.6, 29.7, 29.1, 29.0, 28.8, 27.9, 27.3, 22.6, 18.7, and 15.4.

In step 4, the quinolone was added to a mixture of 5% Pd/CaCO_3_ and EtOAc, and the mixture was stirred for 20 min at room temperature. A solution of the alkyne in EtOAc was added, and the reaction mixture was stirred for 5 h under a balloon of H_2_. After this period of time analysis of the reaction mixture by TLC (10% EtOAc in hexanes, visualization with KMnO_4_) indicated complete consumption of starting material. The mixture was diluted with 1-M HCl and extracted with EtOAc. The organic extracts were combined and dried (MgSO_4_). The drying agent was removed by filtration. Silica gel was added to the filtrate, and the mixture was concentrated to dryness under reduced. Flash column chromatography (RediSepR_f_, SiO_2_, 100% hexane → 10% EtOAc in hexane, visualizing fractions with KMnO_4_) gave the product as a pale transparent oil (1.7 g, 88%). ^1^H NMR (400 MHz, CDCl_3_): *δ* 5.40–5.35 (m, 2H), 3.65 (t, *J* = 6.0 Hz, 2H), 2.14–2.00 (m, 4H), 1.62 (m, *J* = 7.6 Hz, 2H), 1.50 (m, *J* = 7.6 Hz, 1H), 1.48–1.25 (m, 14H), and 0.86 (d, *J* = 6.0 Hz, 6H). ^13^C NMR (100 MHz, CDCl_3_): *δ* 130.8, 128.8, 62.7, 39.0, 32.6, 29.9, 29.7, 29.6, 29.3, 27.9, 27.4, 27.2, 23.6, and 22.6.

In step 5, solid pyridinium dichromate was added in one portion to an ice-H_2_O bath-cooled solution of the alcohol in anhydrous DMF. The reaction was allowed to warm to room temperature as the cooling bath melted with stirring for 16 h. Analysis of the reaction by TLC (10% EtOAc in hexane, KMnO_4_ stain) after that period of time indicated complete consumption of the starting material. The reaction was diluted into a solution of 10% citric acid. The mixture was extracted with MTBE. The combined organic extracts were washed with saturated aqueous NaCl and then added to silica gel. The resulting mixture was concentrated to dryness under reduced pressure. Flash column chromatography (RediSepR_f_, SiO_2_, 100% hexane → 10% EtOAc in hexanes, fractions visualized with KMnO_4_ stain) gave the product as a clear colorless oil (0.54 g, 30% yield). ^1^H NMR (500 MHz, CDCl_3_): *δ* 5.43 (m, 1H), 5.34 (m, 1H), 2.39 (m, 3H), 2.04 (q, *J* = 7.2 Hz, 2H), 1.50 (m, *J* = 6.8 Hz, 1H), 1.37–1.24 (m, 10H), 1.15 (m, 2H), and 0.86 (d, *J* = 6.8 Hz, 6H). ^13^C NMR (125 MHz, CDCl_3_): *δ* 178.6, 131.9, 126.9, 39.0, 29.9, 29.6 (two signals: 29.62 and 26.59), 29.3, 28.0, 27.4, 27.2, 22.7, and 22.5 (Fig. S4 and S5).

### Pathogen strains and MIC testing

Ampicillin (CAS No. 69–52-3) was purchased from Fisher Scientific. Chloramphenicol (CAS No. 56–75-7) was purchased from MP Biomedical. CCCP (CAS No. 555–60-2), ciprofloxacin (CAS No. 85721–33-1), erythromycin (CAS No. 114–07-8), gentamicin (CAS No. 1405–41-0), rifampicin (CAS No. 13292–46-1), and valinomycin (CAS No. 2001–95-8) were purchased from Millipore Sigma. Daptomycin (CAS No. 103060–53-3), colistin (CAS No. 1264–72-8), and tetracycline (CAS No. 64–75-5) were purchased from GoldBio. Calcimycin (CAS No. 52665–69-7), cerulenin (CAS No. 17397–89-6), linoleic acid (CAS No. 60–33-3), monactin (CAS No. 7182–54-9), platensimycin (CAS No. 835876–32-9), and triclosan (CAS No. 3380–34-5) were purchased from Cayman Chemical.

The MIC of (*Z*)-4C-14:1 against five strains of bacterial pathogens (*B. subtilis* ATCC 23857, *E. faecalis* ATCC 29212, *E. coli* ATCC 25922, *Pseudomonas aeruginosa* ATCC 27853, *S. aureus* ATCC 29213, and *S. pneumoniae* ATCC 49619) was determined in triplicate by standard microdilution method in microtiter plates in cation-adjusted Muller-Hinton broth (CAMHB) as defined by the National Committee for Clinical Laboratory Standards (54 – 56). All antibiotics tested were prepared fresh on the day of testing. All strains were allowed to grow at 37°C on freshly streaked tryptic soy broth (BD Bacto) agar plates except *E. faecalis* and *S. pneumoniae*, which were streaked onto tryptic soy broth plates supplemented with 5% defibrinated sheep blood. *S. pneumoniae* was always grown at 37°C supplemented with 5% CO_2_. The following day, single colonies for each strain were inoculated into 10 mL of CAMHB and grown at 37°C, 120 rpm until the desired OD_600_ was reached equivalent to 1.5 × 10^8^ CFU/mL corresponding to 0.5 McFarland standard. Test compounds were prepared in serial twofold dilutions across the 96-well plate with final concentrations ranging from 0.06 to 128 µg/mL. The culture was diluted 100-fold in CAMHB to reach a concentration of 1.5 × 10^6^ CFU/mL, and 10 µL of this inoculum was added to 90 µL of test compounds in CAMHB, yielding a final volume of 100 µL. Plates were incubated at 37°C for 18 h (or 22 h with 5% CO_2_ for *S. pneumoniae*), and the MIC value was defined as the lowest concentration producing no visible growth.

*B. subtilis* ATCC 23857 was used in the BCP assay due to the bacterium’s large size and susceptibility to (*Z*)-4C-14:1. The growth dynamics of *B. subtilis* (Fig. S6) were determined as follows. A single colony of *B. subtilis* was used to inoculate 10-mL CAMHB and grown at 37°C, 120 rpm for approximately 4 h until an exponential phase was reached (OD_600_ > 0.1). The culture was diluted in CAMHB to an OD_600_ = ~0.02 in a final volume of 60 mL. The OD_600_ of this culture was monitored for over 8 h using a spectrophotometer. At each timepoint, the concentration of bacteria in CFU/mL was determined by plating 100 µL of diluted bacterial culture on LB agar in triplicate. CFU/mL was then calculated using the equation:


$$\frac{CFU}{mL}=\frac{Dilution\;Factor\;\times \;Average\;Number\;of\;Distinct\;Colonies}{The\;amount\;of\;culture\;plated\;\left(mL\right)}$$

The MICs for 17 antibiotics were determined for *B. subtilis* ATCC 23857 (Table S1) by standard microdilution method in microtiter plates in CAMHB as described above. All antibiotics tested were prepared fresh on the day of testing. Briefly, *B. subtilis* was streaked on LB agar plate, and a single colony was inoculated in 10-mL CAMHB and allowed to grow at 37°C, 120 rpm for 3.5 h until the OD_600_ reached 0.497, equivalent to 1.5 × 10^8^ cells/mL. Antibiotic compounds were prepared in serial twofold dilutions across the 96-well plate with final concentrations ranging from 0.006 to 256 µg/mL. The 96-well plate was incubated for 18 h at 37°C, and the MIC value was defined as the lowest concentration producing no visible growth.

### Fluorescence microscopy

*B. subtilis* was streaked on an LB agar plate and incubated overnight at 32°C for 18 h. Single colonies were used to inoculate 10-mL CAMHB and incubated at 37°C, 120 rpm overnight. Overnight, cultures were then diluted in CAMHB to an OD_600_ 0.100 and incubated at 37 °C, 120 rpm for ~1.5 h until OD_600_ 0.195, equivalent to 3 × 10^7^ cells/mL (exponential growth). Exponential phase cells in 1-mL aliquots were then treated at 2× or 5× MIC concentrations (Table S2) at 37°C on a rotator until distinct morphology changes in the cells were observed ranging between 30 min and 6 h (13). For the BCP assay, an incubation time of only 30 min for colistin at 5× MIC was deemed the maximum time of exposure of *B. subtilis* to this antibiotic due to significant cell loss observed. Moreover, incubation times longer than 2–3 h in the BCP assay were needed for selecting antibiotics in order to obtain a distinct cell morphology compared to untreated control cells.

A tri-dye mixture containing FM4-64 (558/734 nm) (Invitrogen), SYTOX Green (504/523 nm) (Invitrogen), and DAPI (360/460 nm) (Invitrogen) were made prior to imaging samples. Lyophilized 100-µg vials of FM4-64 were stored at −20°C and resuspended in 100-µL sterile DMSO to obtain a 1,000-µg/mL stock. A stock of 5,000-µg/mL DAPI was made by adding 2 mL of nuclease-free H_2_O to 10-mg DAPI and stored at −20°C. A 500-µM stock of SYTOX Green was made by adding 10 µL of 5-mM SYTOX Green stock to 90 µL of sterile DMSO and stored at −20°C. A 300-µL tri-dye mix contained 286.5-µL sterile filtered 1× PBS, 9 µL of 1,000-µg/mL FM-464, 1.5 µL of 500-µM SYTOX Green, and 3 µL of 5,000-µg/mL DAPI.

Bacterial samples in 1-mL aliquots were centrifuged for a minute at 6,000 rpm, 900 µL of supernatant was removed, and the cell pellet was resuspended in 100 µL of remaining CAMHB. A 12-µL aliquot of resuspended cells was incubated with 3 µL of tri-dye mix for 20–25 min at room temperature in the dark. The final concentrations of the dyes within the sample mixture were 6-µg/mL FM4-64, 0.5-µM SYTOX Green, and 10-µg/mL DAPI. The samples were then pipetted onto a glass coverslip affixed to a 35-mM diameter dish and covered with 1.2% ultrapure agarose (Invitrogen) pads to immobilize cells (20) and immediately imaged.

Cells were imaged using a Zeiss Axio Observer seven equipped with Zeiss Axiocam 702 monochrome CMOS and Zeiss Axiocam 503 color CCD cameras using the oil immersion 100× objective lens at the Cell and Developmental Biology Microscopy Core within the University of Pennsylvania. Images were taken in *z*-stacks with 13 slices measuring 0.24-µm thick. Raw images were deconvolved using the imaging program Zeiss ZEN Blue constrained iterative deconvolution software (version 2.5.75.6). GPU processing accelerations were used during deconvolution, and fluorescence decay corrections were applied to all deconvolved images. The median focal plane is shown. Imaging parameters and exposure times for each dye were kept constant (DAPI was imaged using a 385-nm LED line with an exposure time of 100 ms, SYTOX Green was imaged using a 475-nm LED line with an exposure time of 250 nm, and FM4-64 was imaged using a 555-nm LED line with an exposure time of 300 nm). For each antibiotic or untreated sample, four replicates were prepared and imaged. Each sample replicate contained a minimum of 100 cells that were processed for quantitative measurements. All deconvolved and non-deconvolved images were saved in the CZI format.

### Generation of cell morphology measurements

Non-deconvolved images were imported into ImageJ Fiji 2 (2.3.0 version), and the FM4-64 channel was used to determine the medial focal plane using the Focus LP plug-in (1.0.x version). TIFFs were created in Fiji of the focused slice for all three channels for both non-deconvolved and deconvolved images. Images were then analyzed using CellProfiler (version 4.2.5) (57). Cells were initially identified using the deconvolved FM4-64 images, and the objects were expanded using the phase image as a guide to obtain final cell objects. Nucleoids were identified separately and associated with corresponding cells. Cells were then filtered based on the presence of nucleoids in the deconvolved DAPI channel, and only cells with DAPI fluorescence were included in the analysis. Images were run through a pipeline (File S1) that quantified 39 separate cell morphological and fluorescence-based parameters per cell. The measured parameters used for analysis and their definitions are listed in Table S3. Each replicate yielded at minimum 100 cells from which quantitative parameters were generated. Fluorescence intensity measurements were measured only on non-deconvolved images. All CellProfiler data were exported and saved as a CSV file.

### Principal component analysis

Individual cells with missing data across the 39 morphological parameters were removed, and cells with values over five standard deviations from the within-treatment mean for any of the 39 parameters were removed. To weigh each treatment identically, 217 cells were randomly selected from each treatment, as this was the lowest number of cells available in a single treatment (File S2). PCA was calculated for pairwise sets of (*Z*)-4C-14:1-treated cells and each antibiotic-treated cells using R’s prcomp function, with morphological parameters scaled and centered. The first two principal components and 95% confidence ellipse for each treatment were graphed using ggplot2. We calculated pairwise concordance as the number of cells found in both 95% confidence ellipses divided by the total number of cells in either of the 95% confidence ellipses. This analysis was completed using R v4.2.1, RStudio v2022.07.01 Build 554, and the following packages: broom v1.0.4, ggplot2 v3.4.1, gridExtra v2.3, readr v2.1.4, and tidyverse v2.0.0.

### Cytotoxicity assay

The cytotoxicity assay was performed using a human liver cell line (HepG2, ATCC HB-8065) cultured according to methods described in Donato et al. (58). Briefly, cell cultures were grown at 37℃/5% CO_2_ in Dulbecco’s Modification of Eagle’s Medium (DMEM) supplemented with 10% fetal bovine serum (FBS), with final concentrations of 4-mM penicillin/streptomycin and 50-U/mL L-glutamine, and passaged every 6 days at a ratio of 1:6. Cell viability was determined via the trypan blue exclusion method (59) and averaged 96% viability before all experiments were performed.

The cytotoxicity of (*Z*)-4C-14:1 was evaluated in HepG2 cells with the vehicle DMSO and Triton X (0.1%) as the negative control and positive control, respectively. Briefly, after log-phase growth, HepG2 cells were seeded at 2 × 10^4^ cells/well in a 96-well microtiter plate and left overnight to establish attachment. Following attachment, the media was removed, and cells were dosed in triplicate wells for 48 h with a range of (*Z*)-4C-14:1 concentrations (1–2,218 µM of final concentrations), DMSO (0.9%) or Triton X. Viability of cells was evaluated using a commercial ATP assay (CellTiterGlo 2.0, Promega, Madison, WI, USA) where 100 µL of CellTiterGlo 2.0 reagent was added to each well containing 100 µL of media and cells and then incubated for 10 min, after which 100 µL of the well contents was transferred to a microfuge tube and luminescence was measured on the Promega Glomax 20/20 using 1-s integration. Each assay was repeated five times and viability measurements were normalized to paired vehicle controls and used to calculate the IC_50_ value by nonlinear regression using a variable slope with four parameters in Graphpad Prism (version 9.5.1).

### Diagnostic fluorescence staining of cells treated with (*Z*)-4C-14:1

FM4-64 (6 µg/mL) and WGA-Alexa Fluor 488 (Molecular Probes) (100 µg/mL), which stain the cell membrane and peptidoglycan layer, respectively, were used to stain *B. subilis* cells exposed to (*Z*)-4C-14:1 at 2× MIC for 2 h or untreated cells. WGA is a lectin that binds to oligomers of *N*-acetylglucosamine and *N*-acetylmuramic acid (60). *B. subtilis* cells were grown, stained, and mounted as described above. Cells were imaged on a Leica Stellaris 5 DMi8 series laser scanning confocal microscope equipped with Power HyD S detectors and 63× oil objective.

## Data Availability

The strain A414 was identified as *Olleya marilimosa* (GenBank accession number OP950677).

## SUPPLEMENTAL MATERIAL

Supplemental material jb.00310-23-s0001.pdf DOI links to data sets, supplemental Tables, and supplemental figures.

ASM does not own the copyrights to Supplemental Material that may be linked to, or accessed through, an article. The authors have granted ASM a non-exclusive, world-wide license to publish the Supplemental Material files. Please contact the corresponding author directly for reuse.
