# Supplementary material for: A marine-derived fatty acid targets the cell membrane of Gram-positive bacteria
Source: J Bacteriol. 2023 Oct 31;205(11):e00310-23. doi: 10.1128/jb.00310-23 (PMC10662121; doi:10.1128/jb.00310-23)
Supplement: Supplemental material — DOI links to data sets, supplemental Tables, and supplemental figures. [file jb.00310-23-s0001.pdf]

SUPPLEMENTAL MATERIAL

A Marine Derived Fatty Acid Targets the Cell Membrane of Gram-Positive Bacteria

Authors: Isha Upender[a‡], Olivia Yoshida[a‡], Anna Schrecengost[a], Hilary Ranson[b], Qihao Wu[b], David C. Rowley[b], Shreya Kishore[a], Claire Cywes[a], Eric L. Miller[a], and Kristen E. Whalen[a#]

Author Affiliations:
[a]Department of Biology, Haverford College, Haverford, PA, USA
[b]Department of Biomedical and Pharmaceutical Sciences, College of Pharmacy, University of Rhode Island, Kingston, RI, USA

#Corresponding Author: Kristen E. Whalen, kwhalen1@haverford.edu
[‡]These authors contributed equally. Order was determined alphabetically.

Supplemental Data Files

Supplemental Data File 1. CellProfiler pipeline describing all 39 parameters used to quantitatively describe cell morphology. Access the pipeline at DOI:10.6084/m9.figshare.23808699.

Supplemental Data File 2. Bacterial cell morphology measurements. Excel document containing CellProfiler output data on a per cell basis for each measurement of the 39 parameters used in the BCP analysis. The data are organized in tabs by treatment (antibiotic, untreated, or (*Z*)-4C-14:1). Access data at DOI: 10.6084/m9.figshare.23808675

Supplemental Figures

Supplemental Figure 1. Isolation scheme of (*Z*)-13-methyltetra-4-decenoic acid using bioassay guided fractionation of the crude extract from 30 L volumetric equivalent of *Olleya marilimosa* (A414). Masses of each fraction and percentage mass of the total crude extract are shown.

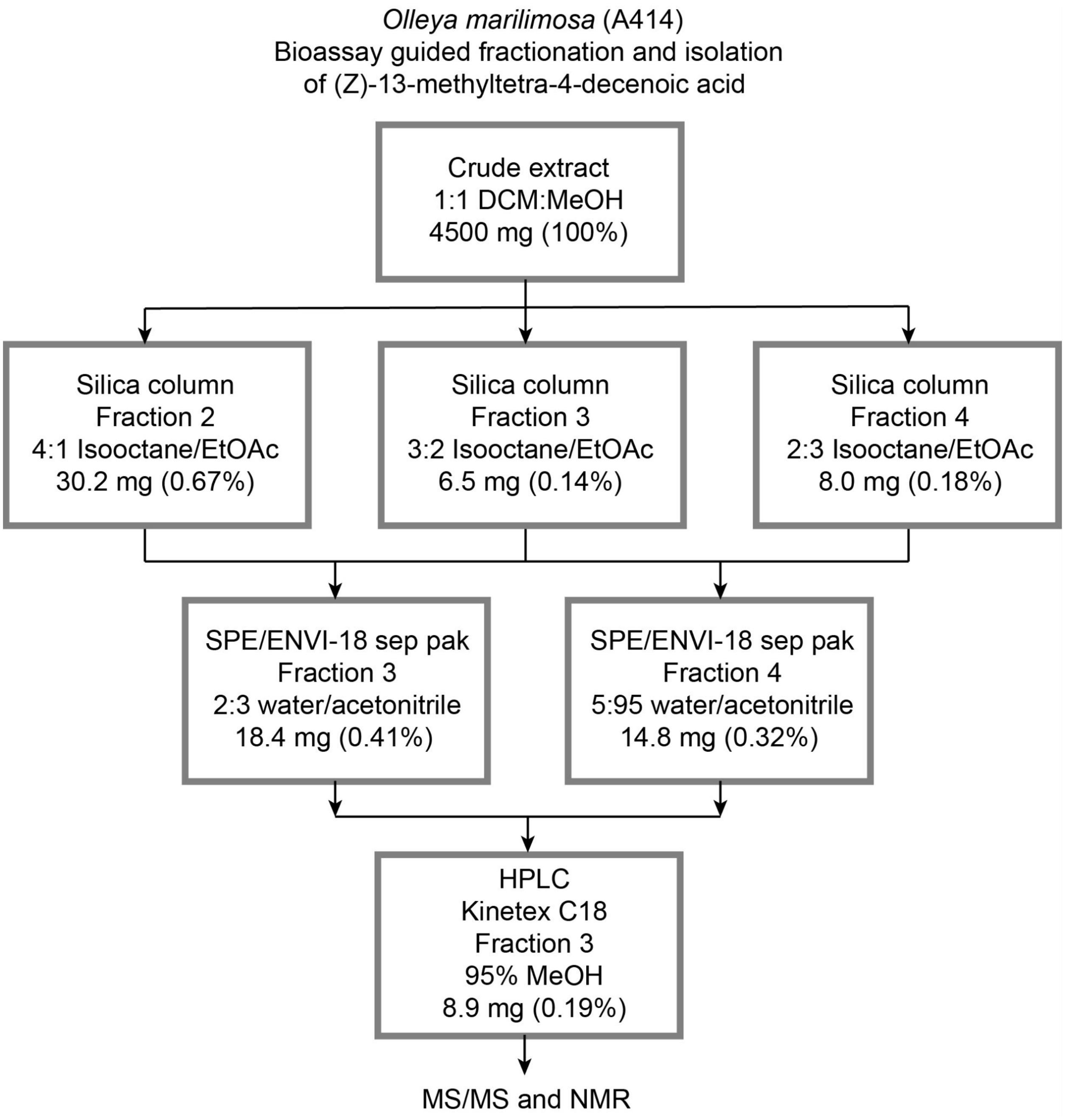

Supplemental Figure 2. ^{1}H, ^{13}C, COSY, HMBC, HSQC NMR of (*Z*)-13-methyltetra-4-decenoic acid. 1D (^{1}H and ^{13}C) and 2D (COSY, HSQC, and HMBC) were measured on 500 MHz Varian Inova NMR spectrometer, and the chemical shifts were recorded as d values (ppm) referenced to solvent residual signals [DMSO-d_6 (δ_H 2.50 ppm, δ_C 39.520 ppm)].

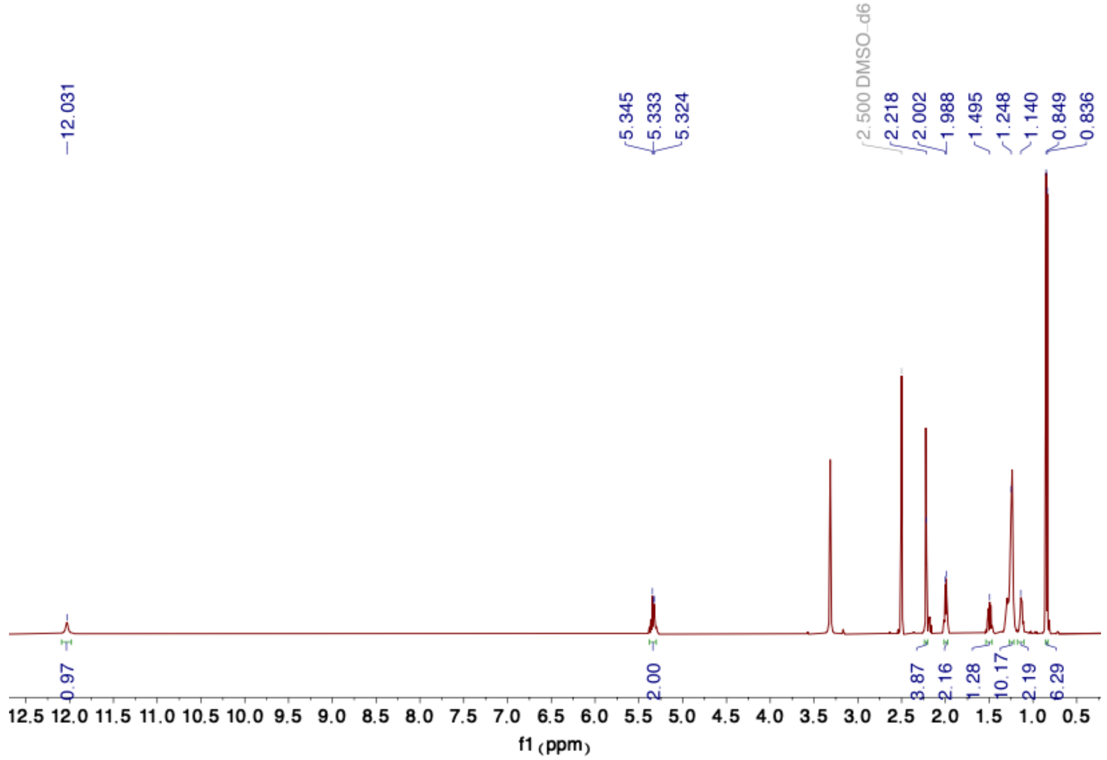

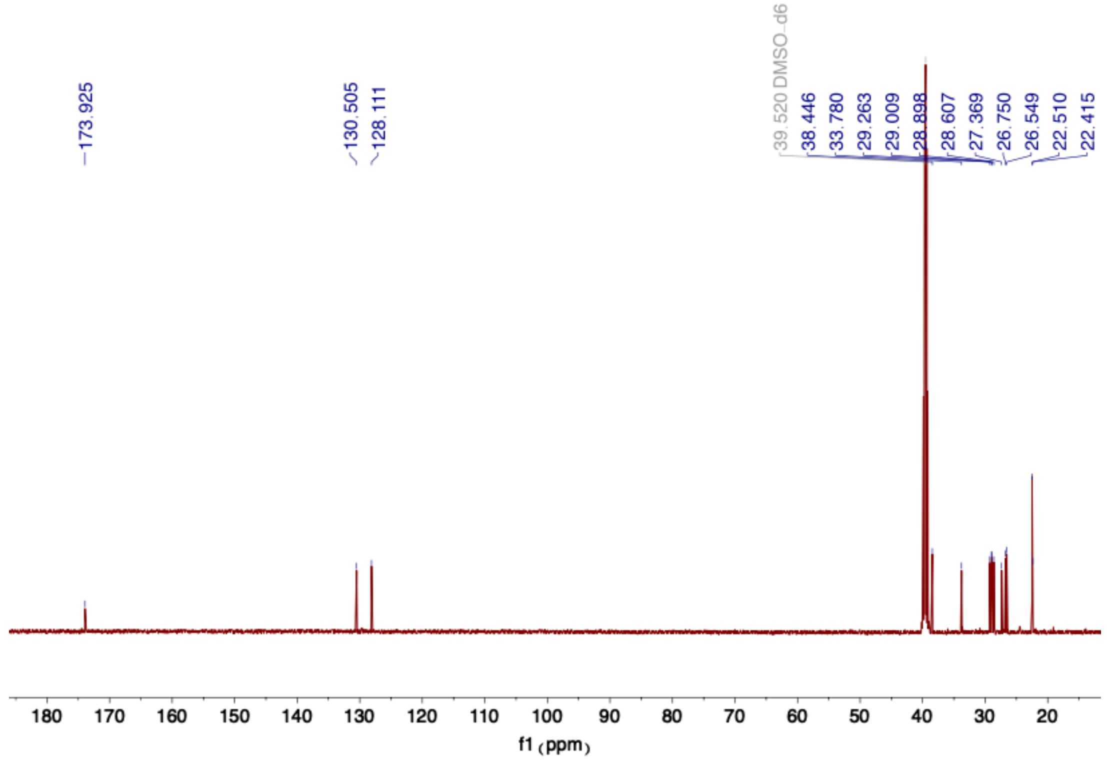

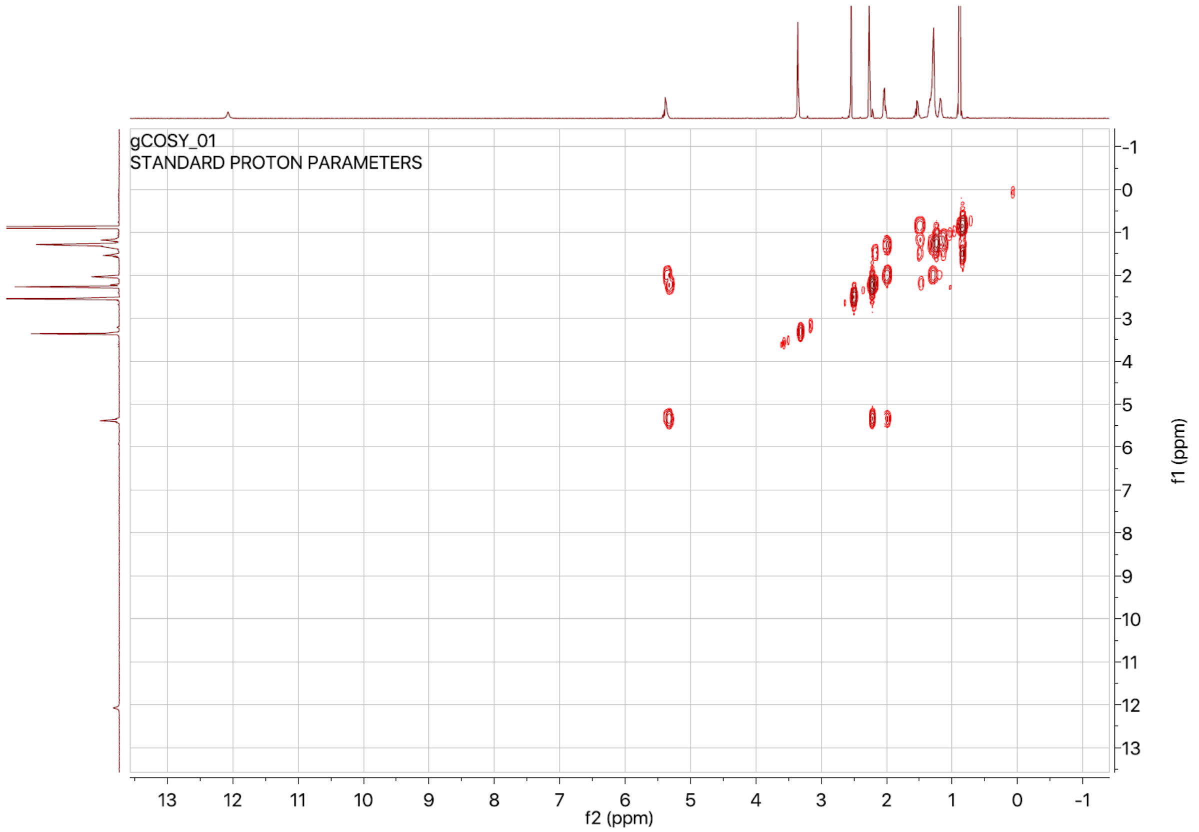
gCOSY_01
STANDARD PROTON PARAMETERS
f2 (ppm)
f1 (ppm)

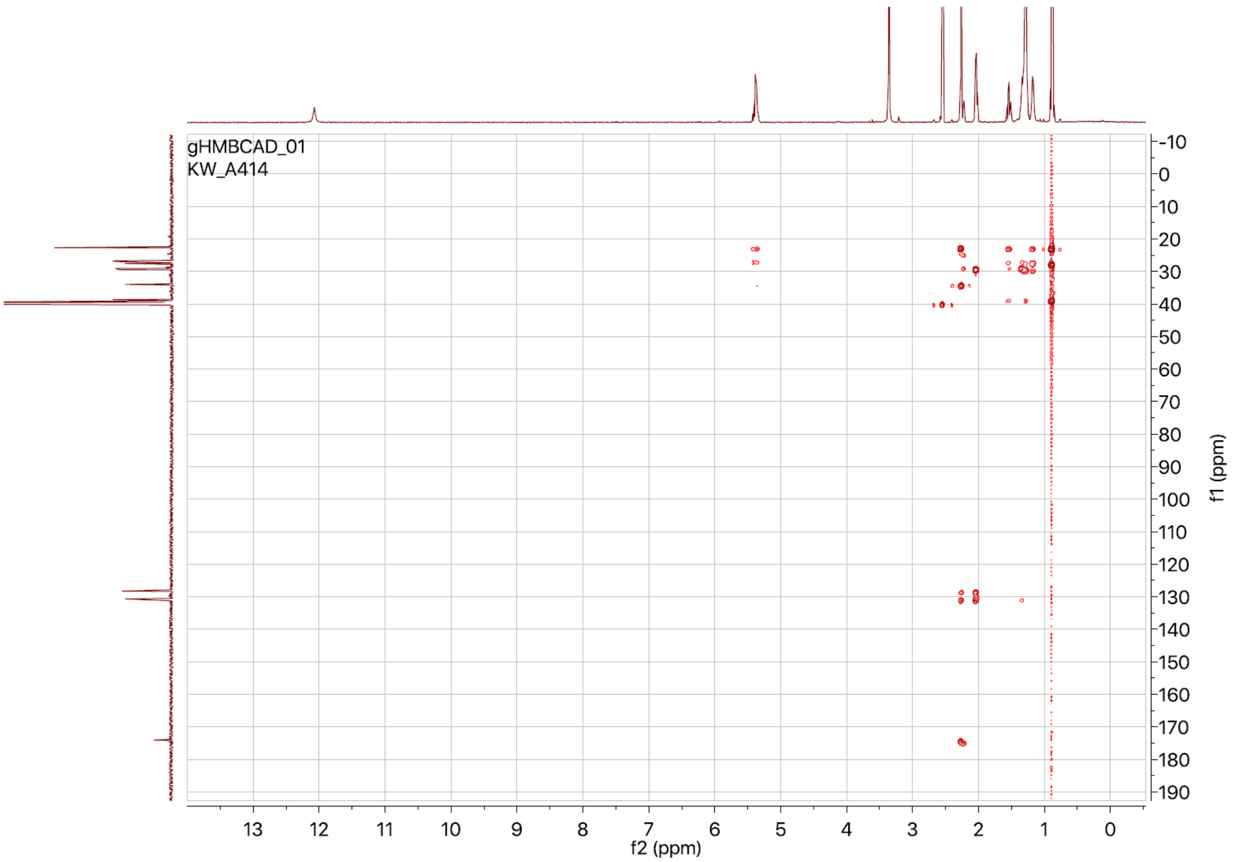
gHMBCAD_01
KW_A414
f2 (ppm)
f1 (ppm)

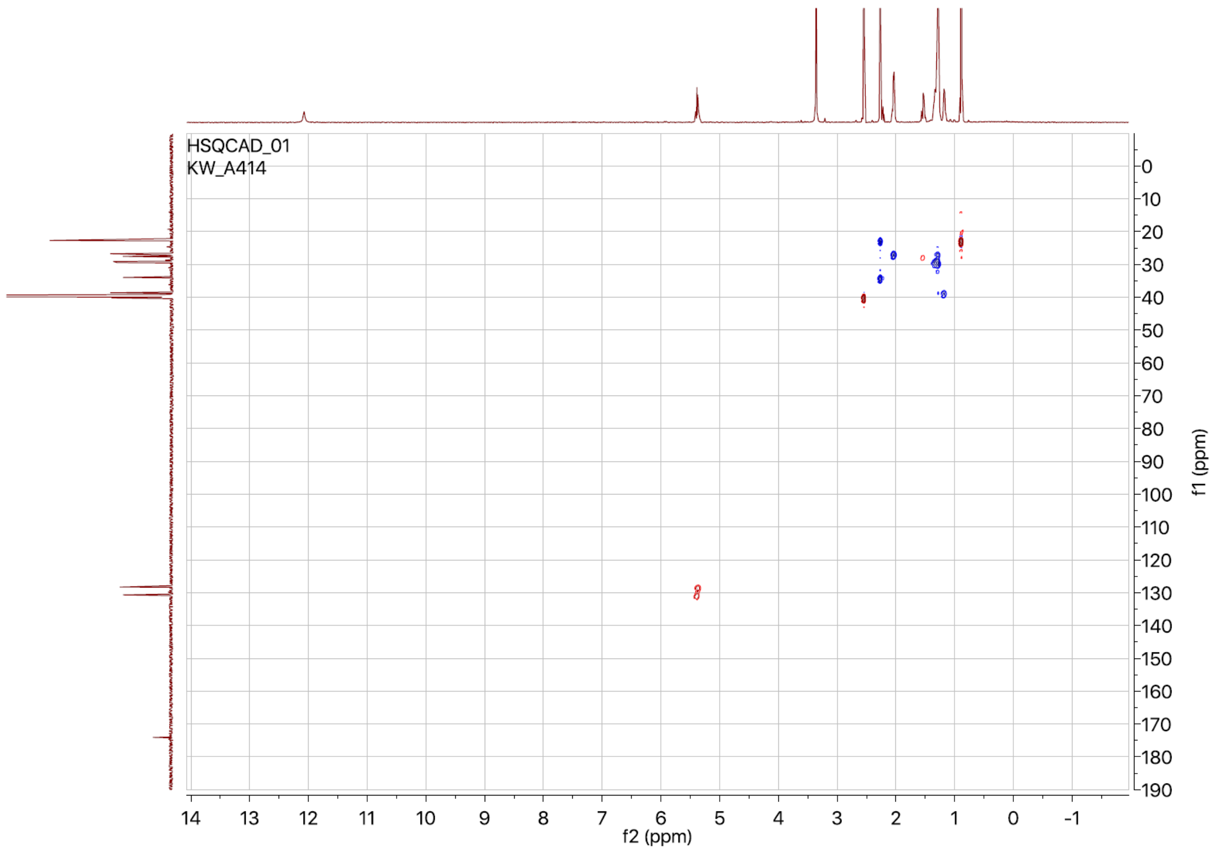
HSQCAD_01
KW_A414
f1 (ppm)
f2 (ppm)

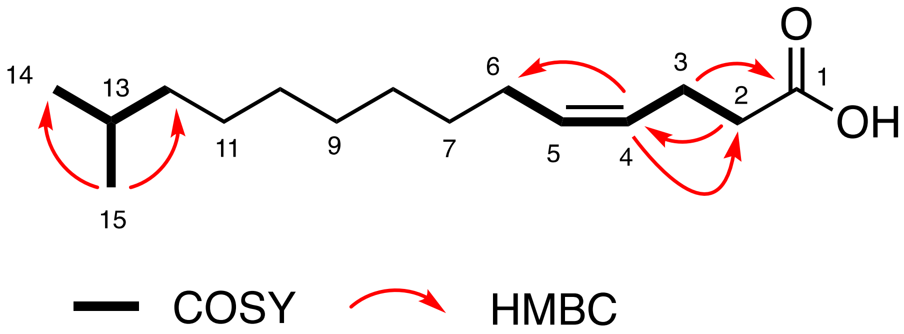
O
OH
1
2
3
4
5
6
7
9
11
13
14
15
COSY
HMBC

Supplemental Figure 3. Synthesis of (*Z*)-13-methyltetra-4-decenoic acid.

Step 1:

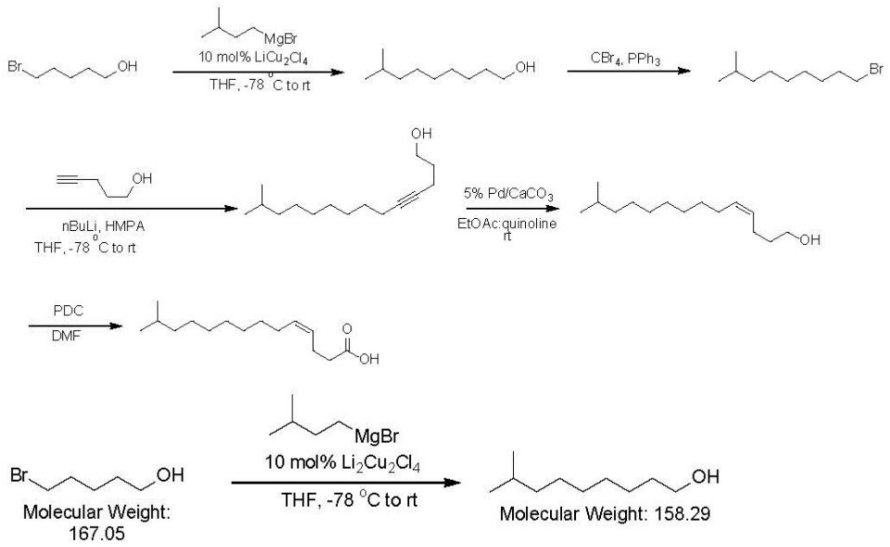

Step 2:

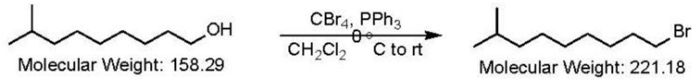

Step 3:

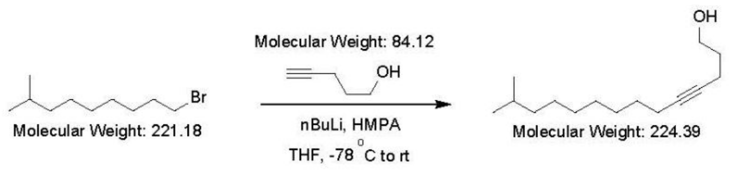

Step 4:

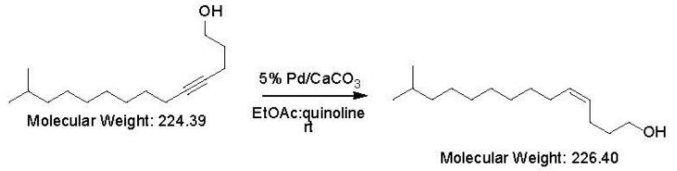

Step 5:

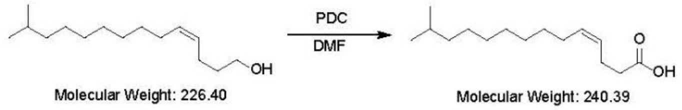

Supplemental Figure 4. ^{1}H NMR of synthetic (*Z*)-13-methyltetra-4-decenoic acid.

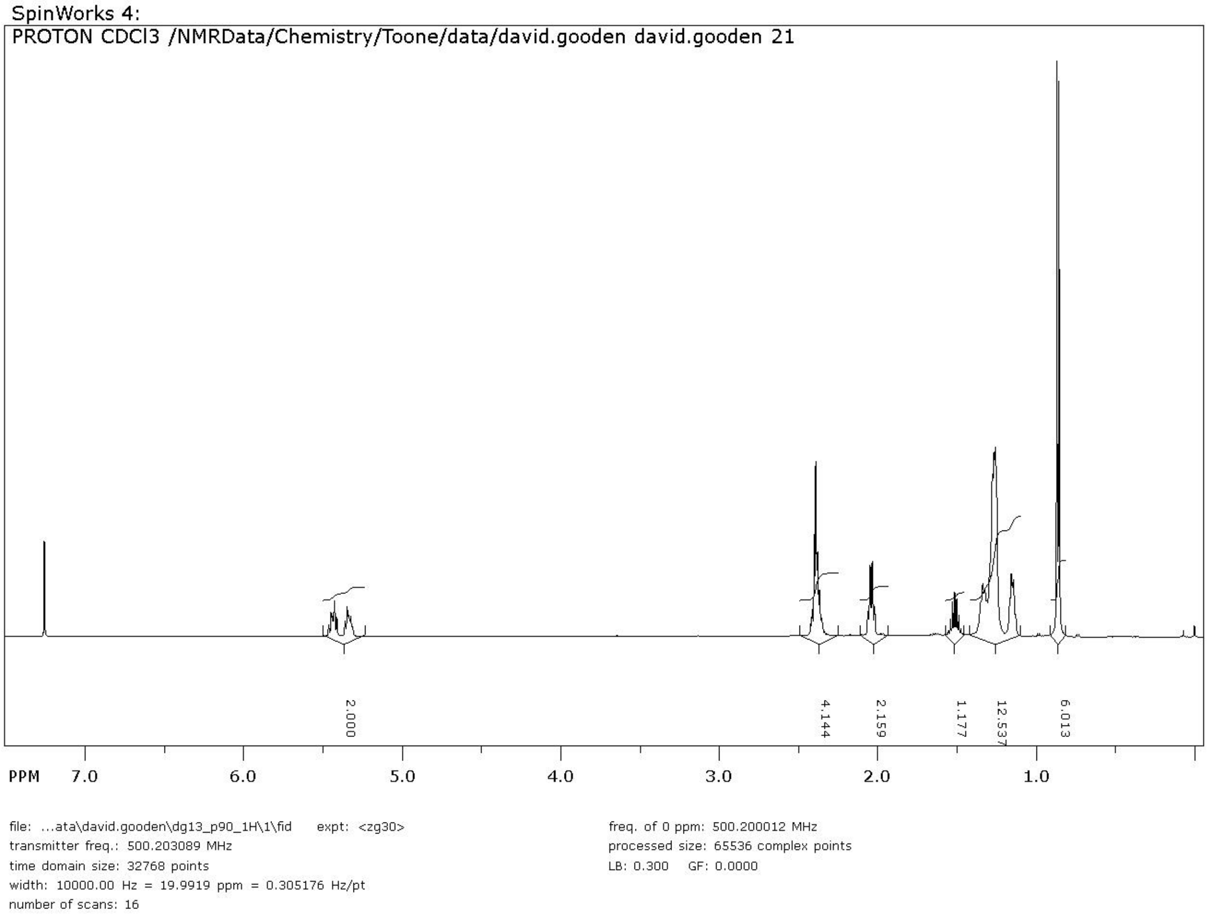

^{1}H NMR of synthetic (*Z*)-13-methyltetra-4-decenoic acid (500 MHz, $CDCl_3$)

Supplemental Figure 5. ^{13}C NMR of synthetic (*Z*)-13-methyltetra-4-decenoic acid.

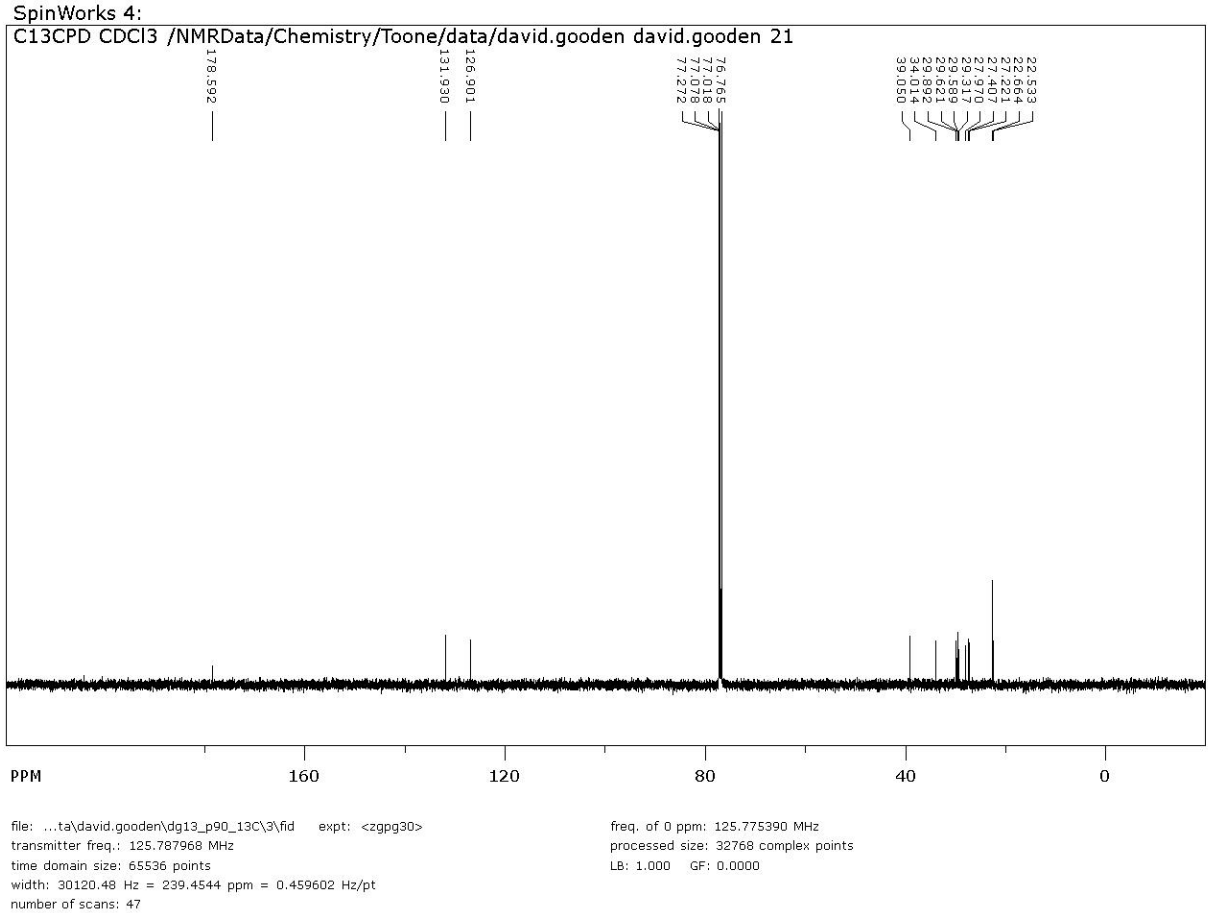

^{13}C NMR of synthetic (*Z*)-13-methyltetra-4-decenoic acid (125 MHz, $CDCl_3$)

Supplemental Figure 6. *Bacillus subtilis* growth curve.

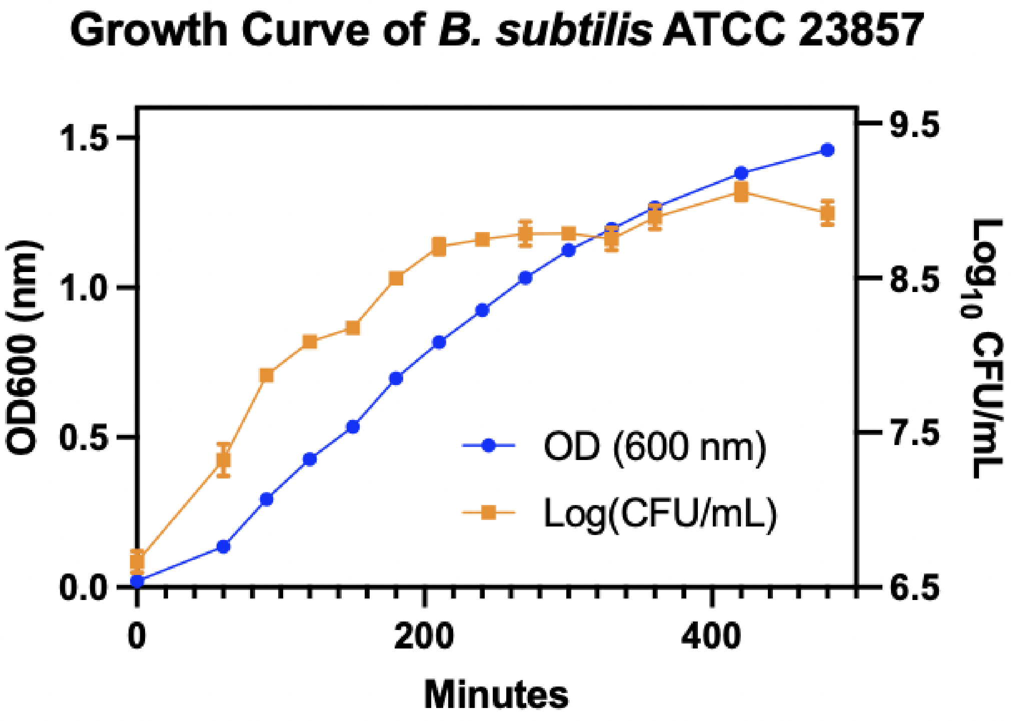

Supplemental Figure 6. *Bacillus subtilis* ATCC 23857 growth was measured in optical density at 600 nm and log CFU/mL (mean ± std. dev. of triplicate readings) over 480 minutes.

Supplemental Tables

Supplemental Table 1. Experimentally determined MICs for antibiotics against *B. subtilis* (ATCC 23857) used in Bacterial Cytological Profiling Assay.

**Supplemental Table 1**	
**Treatment**	**MIC (µg/mL)**
Ampicillin	0.0625
Calcimycin	0.25
CCCP	1
Cerulenin	8
Chloramphenicol	4
Ciprofloxacin	0.125
Colistin	32
Daptomycin	2
Erythromycin	0.06
Gentamicin	0.25
Linoleic Acid	256
Monactin	2
Platensimycin	4
Rifampicin	0.25
Tetracycline	2
Triclosan	2
Valinomycin	≥256

Supplemental Table 2. Antibiotic Treatment Conditions of *B. subtilis* (ATCC23857) in the Bacterial Cytological Profiling Assay.

**Supplemental Table 2**				
**Antibiotic**	**Solvent**	**1x MIC (µg/mL)**	**5x MIC (µg/mL)**	**Incubation time (hrs)**
(*Z*)-4C-14:1	DMSO	32	160	2
Ampicillin	Water	0.0625	0.3125	5-6
Calcimycin	DMSO	0.25	1.25	2
CCCP	100% Ethanol	1	5	4
Cerulenin	100% Ethanol	8	40	4
Chloramphenicol	100% Ethanol	4	20	5-6
Ciprofloxacin	0.1 M HCl	0.125	0.625	6
Colistin	1x PBS	32	160	0.5
Daptomycin	1x PBS	2	10	2
Erythromycin	100% Ethanol	0.06	0.3	5-6
Gentamicin	Water	0.25	1.25	5-6
Linoleic Acid	100% Ethanol	256	1280	2
Monactin	DMSO	2	10	3
Platensimycin	DMSO	4	20	6
Rifampicin	DMSO	0.25	1.25	5-6
Tetracycline	70% Ethanol	2	10	5-6
Triclosan	100% Ethanol	2	10	2
Valinomycin	DMSO	≥256	512* (2× MIC)	2

Footnote: *indicates those antibiotics that had to be tested at a concentration < 5× MIC in the BCP assay.

Supplemental Table 3. CellProfiler Module Description of 39 Parameters Used to Quantitatively Describe Fluorescent Images. Descriptions adapted from CellProfiler 4.05 documentation (Carpenter et al. 2006, Janssen et al. 2022) unless otherwise specified.

References

Janssen AFJ, Breusegem SY, Larrieu D. Current Methods and Pipelines for Image-Based Quantitation of Nuclear Shape and Nuclear Envelope Abnormalities. Cells. 2022 Jan 20;11(3):347. doi: 10.3390/cells11030347. PMID: 35159153; PMCID: PMC8834579.

Carpenter, A. E. et al. CellProfiler: image analysis software for identifying and quantifying cell phenotypes. Genome Biol. 7, R100 (2006). (CellProfiler version 4.2.5).

**Supplemental Table 3.**	
**Parameters**	**Function**
**DNA Measurements**	
Math_DNA_Area_Micron_squared	The number of pixels in microns squared in the DNA region
Math_DNA_Compactness	Compactness = $\frac{Mean\ squared\ distance\ of\ object's\ pixels\ from\ centroid}{DNA\ area}$ 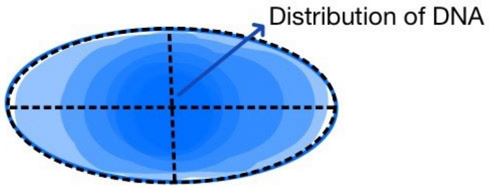
Math_DNA_Eccentricity	The roundness/amount of circular shape in the DNA 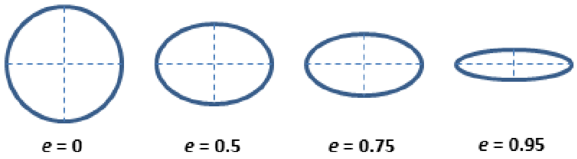 Credit: Carpenter et al., 2006 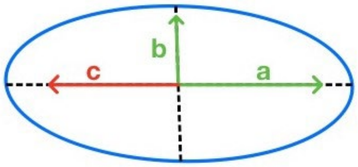

	Eccentricity = $\frac{c}{a}$ with $c^2 = a^2 - b^2$<br>Credit: Janssen et al., 2022
Math_DNA_Extent	The proportion of the pixels in the convex hull that are also in the DNA area region<br><br>Extent = $\frac{area/volume\ ratio\ of\ object}{area/volume\ ratio\ of\ convex\ hull}$<br>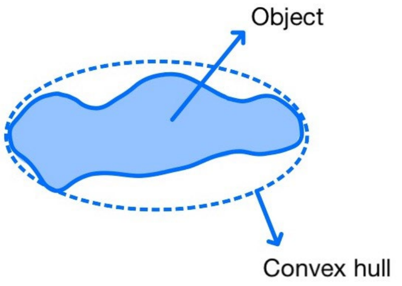
Math_DNA_FormFactor	The ratio of DNA area to DNA perimeter<br><br>Form Factor = $\frac{4\pi * Area}{(Perimeter)^2}$
Math_DNA_MajorAxisLength_Micron	The length in pixels of the major axis of the ellipse that has the same normalized second central moments as the region<br>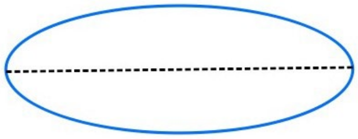
Math_DNA_MaxFeretDiameter_Micron	Feret diameter is the distance between two parallel lines tangent on either side of the object. The maximum Feret diameter is the largest possible diameter, rotating the calipers along all possible angles.<br>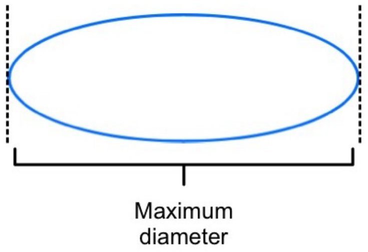
Math_DNA_MaximumRadius_Micron	The maximum distance of any pixel in the object to the closest pixel outside of the object – the largest possible radius of the DNA object
Math_DNA_MeanRadius_Micron	The mean distance of any pixel in the object to the closest pixel outside of the object

Math_DNA_MinFeretDiameter_Micron	Feret diameter is the distance between two parallel lines tangent on either side of the object. The minimum Feret diameter is the smallest and possible diameter, rotating the calipers along all possible angles. 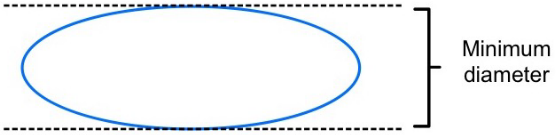
Math_DNA_MinorAxisLength_Micron	The length (in pixels) of the minor axis of the ellipse that has the same normalized second central moments as the region 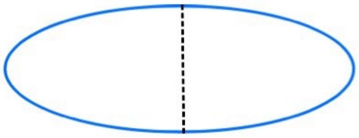
Math_DNA_Perimeter_Micron	The total number of pixels in microns around the boundary of each region in the image
Math_DNA_Solidity	The ratio of convex hull to total area Solidity = $\frac{Object\ area}{Convex\ hull\ area}$
**Membrane Measurements**	
Math_Cell_Area_Micron_squared	The number of pixels, in microns squared, in the cell region
Math_Cell_Compactness	Compactness = $\frac{Mean\ squared\ distance\ of\ object's\ pixels\ from\ centroid}{DNA\ area}$ 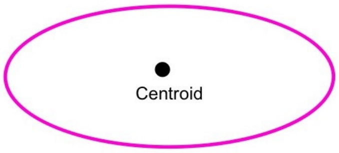
Math_Cell_Eccentricity	The roundness/amount of circular shape in the cell 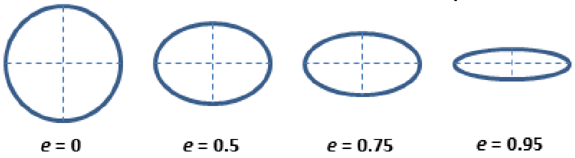 Credit: Carpenter et al., 2006 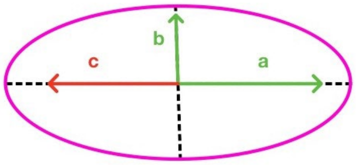

	Eccentricity = $\frac{c}{a}$ with $c^2 = a^2 - b^2$<br>Credit: Janssen et al., 2022
Math_Cell_Extent	The proportion of the pixels (2D) in the convex hull that are also in the cell area region<br><br>Extent = $\frac{area/volume\ ratio\ of\ object}{area/volume\ ratio\ of\ convex\ hull}$<br>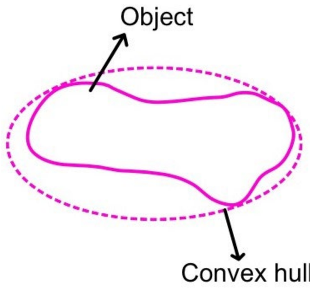
Math_Cell_FormFactor	The ratio of cell area to cell perimeter<br><br>Form Factor = $\frac{4\pi * Area}{(Perimeter)^2}$
Math_Cell_MajorAxisLength_Micron	The length in microns of the major axis of the ellipse that has the same normalized second central moments as the region<br>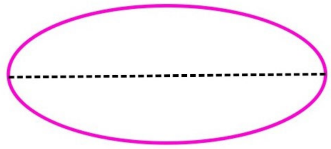
Math_Cell_MaxFeretDiameter_Micron	Feret diameter is the distance between two parallel lines tangent on either side of the object. The maximum Feret diameter is the largest possible diameter, rotating the calipers along all possible angles.<br>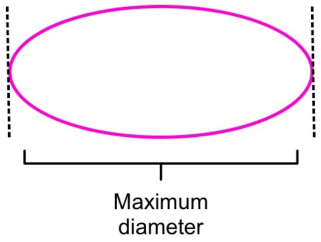
Math_Cell_MaximumRadius_Micron	The maximum distance of any pixel in the object to the closest pixel outside of the object – the largest possible radius of the Cell object

Math_Cell_MeanRadius_Micron	The mean distance of any pixel in the object to the closest pixel outside of the object
Math_Cell_MinFeretDiameter_Micron	Feret diameter is the distance between two parallel lines tangent on either side of the object. The minimum Feret diameter is the smallest and possible diameter, rotating the calipers along all possible angles.<br>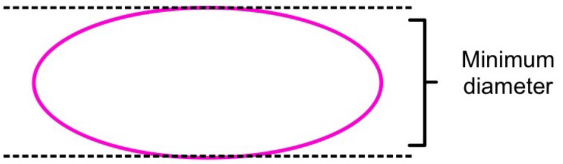
Math_Cell_MinorAxisLength_Micron	The length in pixels of the minor axis of the ellipse that has the same normalized second central moments as the region<br>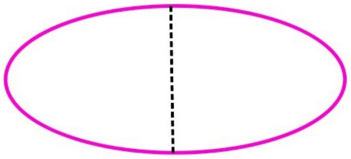
Math_Cell_Perimeter_Micron	The total number of pixels in microns around the boundary of each region in the image
Math_Cell_Solidity	The ratio of convex hull to total area<br>Solidity = $\frac{Object\ area}{Convex\ hull\ area}$<br>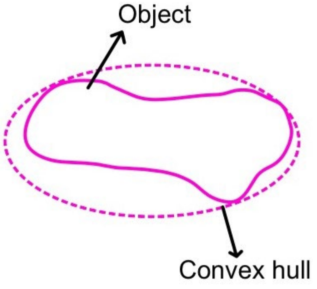
Math_DNA_Per_Cell_Area_Micron_squared	$= \frac{DNA\ area}{cell} * \frac{DAPI\ area}{cell}$
Math_DNA_Per_Cell_MajorAxisLength_Micron	$= \frac{DNA\ area}{major\ cell\ length}$
Math_DNA_Per_Cell_MinorAxisLength_Micron	$= \frac{DNA\ area}{minor\ cell\ length}$

Math_DNA_Per_Cell_Perimeter_Micron	$= \frac{DNA\ area}{cell\ perimeter}$
Math_Decondensation	Decondensation = $\frac{total\ DNA\ area\ per\ cell\ microns\ squared}{cell\ area\ microns\ squared}$
**Fluorescence Intensity Measurements**	
Math_Rescaled_FM464Mask_DAPI_MeanIntensity_per_Cell	The average DAPI fluorescence intensity across all DNA objects in the cell
Math_Rescaled_FM464Mask_DAPI_StdIntensity_per_Cell	The standard deviation of DAPI fluorescence intensity across all DNA objects in the cell
Math_Rescaled_FM464Mask_SytoxG_MeanIntensity_per_Cell	The average SYTOX mean intensity across cell
Math_Rescaled_FM464_MeanIntensity	The average FM4-64 intensity across cell
Math_Total_DNA_Area_Per_Cell_Micron_squared	$= \frac{DNA\ Area}{Cell\ area}$
**Node Measurements**	
Math_Node_Area_Micron_squared	The number of pixels in microns squared in each identified node of the cell membrane 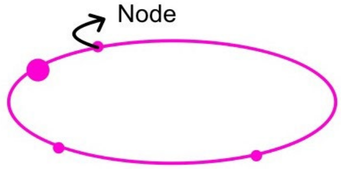
Math_Rescaled_Node_MeanIntensity	The average of FM4-64 intensity across node area
Math_Rescaled_Node_StdIntensity	The standard deviation of FM4-64 intensity across node area
